# Mind the Porins: Differential Effects of Porin Knockouts and Overexpression on Glucose and Xylose Uptake and Utilization in *Pseudomonas putida*

**DOI:** 10.1111/1751-7915.70406

**Published:** 2026-07-03

**Authors:** Barbora Popelářová, Nicolas T. Wirth, Daniel C. Volke, Tibor Botka, Pablo I. Nikel, Pavel Dvořák

**Affiliations:** ^1^ Department of Experimental Biology, Faculty of Science Masaryk University Brno Czech Republic; ^2^ The Novo Nordisk Foundation Biotechnology Research Institute for the Green Transition (BRIGHT) Technical University of Denmark Kongens Lyngby Denmark

**Keywords:** biotechnology, glucose, gram‐negative bacteria, lignocellulose, metabolic engineering, porins, *Pseudomonas putida*, xylose

## Abstract

Porins serve as the primary transport channels for substrate molecules across the outer membrane of Gram‐negative bacteria. Despite their potential to influence substrate uptake in microbial cell factories, porins are often overlooked in metabolic engineering approaches. In this study, we investigate the impact of modulation of sugar porin expression using laboratory and industrial workhorse *Pseudomonas putida* . We first examined the *P. putida* porin repertoire through bioinformatic analysis. Among the two selected porin sets, only the one comprising OprB‐I, OprB‐II and OprB‐III was found to be relevant for glucose catabolism in two biotechnologically important *P. putida* strains. Functional studies involving gene knockouts, complementation and overexpression revealed that the substrate specificity of *P. putida* OprB porins extends beyond glucose and includes the non‐native substrate xylose. Overexpression of *oprB‐I* alone was sufficient to restore sugar utilization in strains with all three *oprB* genes knocked out. Notably, when Gcd was active in *P. putida* , *oprB*‐I overexpression accelerated the utilization of glucose and xylose in mixed sugar conditions through altered sugar uptake and oxidation dynamics. This work exposes the relevance of porins in shaping the uptake of major lignocellulosic sugars and highlights the importance of incorporating outer membrane transport considerations into metabolic engineering strategies for Gram‐negative bacteria.

## Graphical abstract

Altering the levels of outer membrane (OM) porins has an effect on the dynamics of the utilization and co‐utilization of native (glucose—green hexagon) and non‐native (xylose—brown pentagon) sugars, as well as the accumulation of their oxidation byproducts in *Pseudomonas putida* cultures. This work demonstrates the relevance of porins in shaping the uptake of major lignocellulosic sugars and highlights the importance of incorporating outer membrane transport considerations into metabolic engineering strategies for Gram‐negative bacteria.

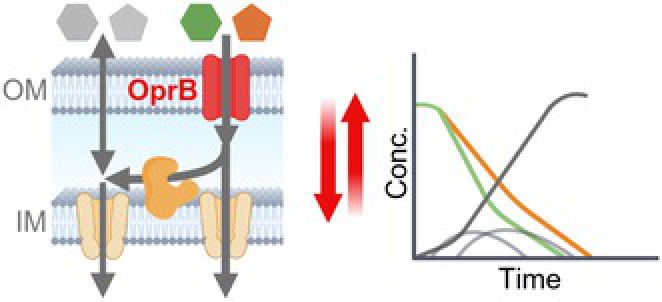

## 1 Introduction

The cell wall of Gram‐negative bacteria spans two phospholipid bilayer membranes and the periplasmic space. The outer membrane is the first semi‐permeable barrier that defines the spectrum of chemicals from the outer environment that can enter the periplasm and later potentially also the cytoplasm of the bacterium (Wang et al. 2021). The lipid component of the outer membrane forms a hydrophobic barrier that limits the passage of polar substances. Passive diffusion of smaller hydrophilic molecules (typically < 600 Da), such as monomeric or oligomeric sugars or some organic acids, is enabled by protein channels called porins. Porins are β‐barrel proteins with a hydrophilic central pore, extracellular loops (that often determine substrate specificity or gating), and short turns on the periplasmic side (Vergalli et al. 2020). Some porins were shown to be more specific towards certain substrates (e.g., OprB for glucose, BenF‐like for benzoate in *Pseudomonas putida* ) (Saravolac et al. 1991; D'Arrigo et al. 2019); others are called general porins and allow passive passage of diverse polar molecules based on a size cut‐off (Welte et al. 1995). Expression of porin genes is often influenced by environmental factors such as nutrient availability, stress or presence of toxins (e.g., cadmium or phenol) (Roma‐Rodrigues et al. 2010; Manara et al. 2012). Porins are thus key components of transport machinery in Gram‐negative bacteria that determine whether and how sugars, organic acids, alcohols and other substrates or chemicals and drugs can pass through (Vergalli et al. 2020).

It is surprising, in this context, how little attention has been dedicated to porins in the fields of microbial metabolic engineering and biotechnology. Rare examples of porins being considered for improving bacterial cell factory properties include the study by Ladkau et al. (2016). In this work, the conversion of renewable dodecanoic acid methyl ester (DAME) to 12‐aminododecanoic acid methyl ester, a building block for the high‐performance polymer Nylon 12, in engineered *Escherichia coli* BL21 (DE3) with an orthogonal biosynthetic route was improved significantly by introducing DAME‐attracting porin AlkL from the alkane degradation operon of *P. putida* GPo1. Löwe et al. (2020) introduced a sucrose utilization gene cluster from *Pseudomonas protegens*, including a ScrY‐like porin, into *P. putida* KT24440, which overcame outer membrane transport limitations and enabled efficient growth on sucrose, the main sugar in waste molasses. Another study identified upregulated BenF‐like porins in *P. putida* KT2440 as important drivers of lignin‐derived ferulic acid utilization (D'Arrigo et al. 2019). Mapping the outer membrane transport and role of porins specifically in *P. putida* is desirable. This robust bacterium is drawing considerable attention as a host for next‐generation industrial biotechnologies and valorization of diverse waste‐derived substrates, including lignocellulosic sugars and aromatics, glycerol, plastic waste components, or even C1 compounds (Silva et al. 2025). The effect of porins on the efficiency of uptake and (co)utilization of these substrates should not be underestimated.

*P. putida* KT2440, as a saprophytic soil bacterium often dwelling in the plant rhizosphere, orients naturally towards the utilization of amino acids, aromatic compounds (including benzoate, *p*‐coumarate and ferulate), or organic acids (for instance, citrate, pyruvate or succinate), and some sugars (such as glucose and fructose) (Belda et al. 2016). Compared to *E. coli* , the outer membrane of *P. putida* is considered less permeable due to the complete absence of general porins (van den Berg 2012). Pseudomonads only encode specific porins, most of which still await detailed characterization (Molina et al. 2020). Among the best described are glucose‐inducible OprB porins—cation‐selective pores that enable the passage of glucose and some other carbohydrates, including sucrose, maltose, arabinose or fructose (Saravolac et al. 1991; Shrivastava et al. 2011). While these sugar‐specific porins have been studied in *P. aeruginosa* (Wylie and Worobec 1995) and in various *P. putida* strains (Saravolac et al. 1991; Shrivastava et al. 2011; van den Berg 2012), in vivo studies in *P. putida* KT2440, which harbours three *oprB* paralogues, *oprB‐I* (*PP_1019*), *oprB‐*II (*PP_1445*) and *oprB‐*III (*PP_3570*), are unavailable.

**FIGURE 1:**
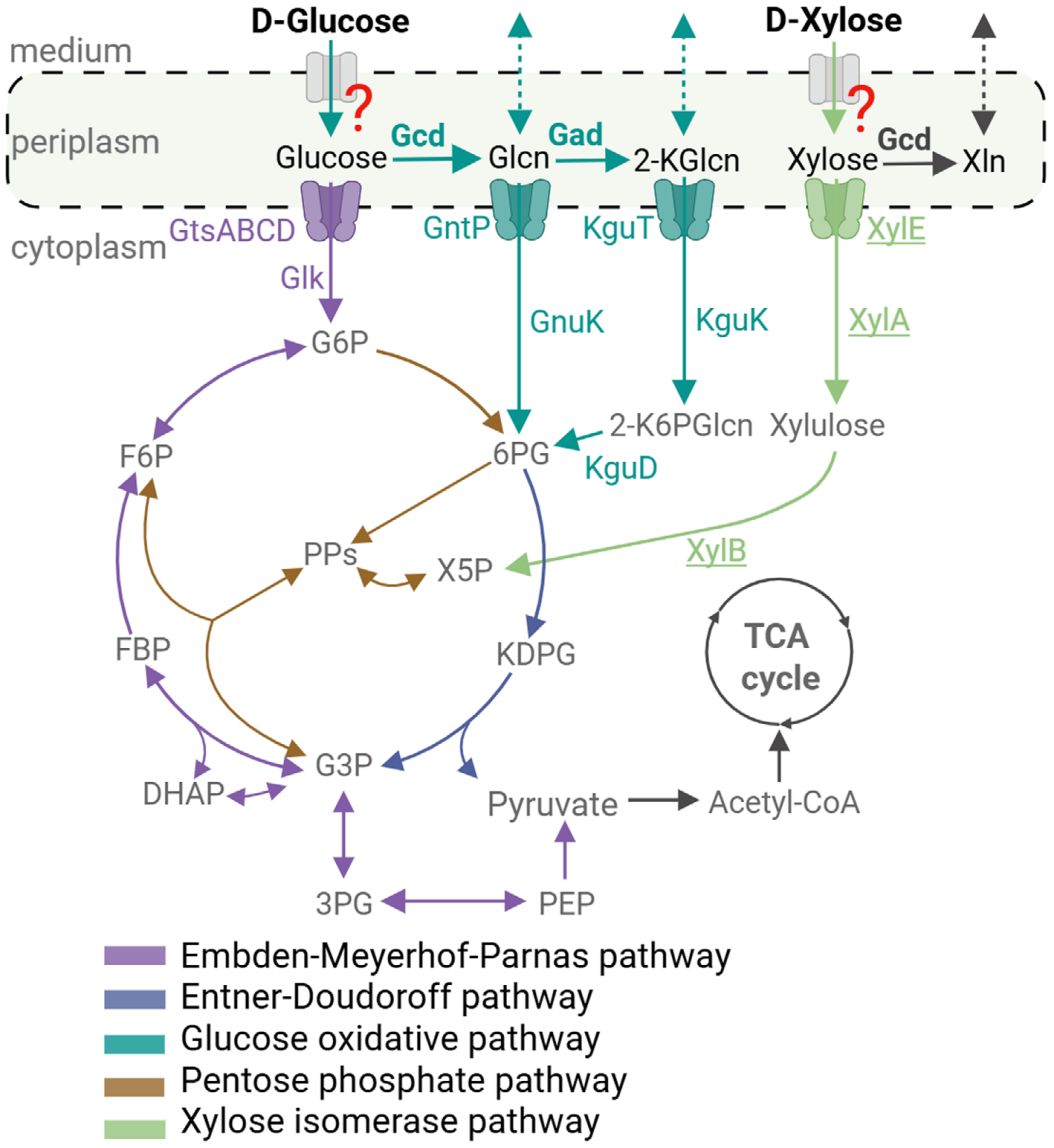
Schematic representation of the upper metabolism of glucose and xylose in *Pseudomonas putida* . Question marks denote transport by porins studied here; dotted arrows show potential diffusion of metabolites across the outer membrane (also by porins, not discussed here). Exogenous XylE, XylA and XylB are underlined. Abbreviations: 2‐KGlcn, 2‐ketogluconate; 3PG, 3‐phosphoglycerate; 6PG, 6‐phosphogluconate; DHAP, dihydroxyacetone phosphate; F6P, fructose‐6‐phosphate; FBP, fructose‐1,6‐biphosphate; G3P, glyceraldehyde‐3‐phosphate; G6P, glucose‐6‐phosphate; Gad, gluconate dehydrogenase; Gcd, glucose dehydrogenase; Glcn, gluconate; Glk, glucokinase; GntP, gluconate permease; GnuK, gluconate kinase; GtsABCD, ABC glucose transporter; KDPG, 2‐keto‐3‐deoxy‐6‐phosphogluconate; KguK, 2‐ketogluconate kinase; KguT, putative 2‐ketogluconate transporter; PEP, phosphoenolpyruvate; PPs, pentose phosphates; TCA cycle, tricarboxylic acid cycle; X5P, xylulose‐5‐phosphate; Xln, xylonate; XylA, xylose isomerase; XylB, xylulose kinase; XylE, xylose‐proton symporter. Note that the Gcd reaction gives rise to gluconolactone or xylonolactone, which is hydrolysed to gluconate or xylonate, respectively, by native lactonase or via spontaneous hydrolysis; this step is not shown. Created by BioRender.com.

Here, we aimed to study the impact of selected porins on the (co)utilization of two prevalent lignocellulosic sugars, glucose and xylose, by *P. putida* (Figure 1). We used two host strains in parallel: strain EM42, a genome‐streamlined derivative of *P. putida* KT2440 (Martínez‐García et al. 2014), and EM42 Δ*gcd* Δ*hexR*, in which the genes encoding periplasmic glucose dehydrogenase (Gcd) and the transcriptional regulator HexR were deleted. The EM42 strain—lacking the flagellar machinery and proviral load—has become a popular chassis in both fundamental and biotechnology‐driven studies (Dvořák, Kováč, and de Lorenzo 2020; Burýšková et al. 2025), while EM42 with inactive Gcd and HexR has emerged as the preferred strain for the (co)utilization and valorization of lignocellulosic carbohydrates, including glucose and xylose (Bujdoš et al. 2023; Dvořák et al. 2024; Ling et al. 2022). The results show that, although the selected porins are not the sole transport mechanisms in the outer membrane of *P. putida* , their manipulation significantly affects sugar uptake dynamics and may have important biotechnological implications.

## 2 Materials and Methods

### 2.1 Candidate Porin Genes Selection

A list of candidate porin‐encoding genes was prepared using the annotations in the *Pseudomonas* database (Winsor et al. 2016) (31 genes), MicroScope (Vallenet et al. 2020) (32 genes) and BioCyc (Karp et al. 2019) (33 genes). Additional genes were added based on high amino acid sequence similarity to one of the annotated genes determined by Protein BLAST (Camacho et al. 2009). Selected genes were subjected to a BLAST search against the entire database to categorize them into families. Protein structures were predicted using Swiss‐model (Waterhouse et al. 2018), trRosetta (Yang et al. 2020) or AlphaFold Server (Abramson et al. 2024) if no structure was available in public depositories. Information on porins in bacteria other than *P. putida* was extracted from BioCyc (Karp et al. 2019) and UniProt (The UniProt Consortium 2025); in some cases, a 3D model was predicted by AlphaFold Server (Abramson et al. 2024).

### 2.2 Cultivation Conditions

*E. coli* cells were cultivated at 37°C in liquid (200 rpm, Unimax 1010, Heidolph) or solid LB agar (Serva; 10 g/L tryptone, 5 g/L yeast extract, 5 g/L NaCl). Chemocompetent *E. coli* cells were prepared using the Mix & Go! *E. coli* transformation kit (Zymo Research). *P. putida* cells were grown at 30°C in otherwise analogous conditions. Wherever needed, antibiotics were added in the liquid or solid medium: ampicillin (Amp; 150 μg/mL for *E. coli* ), kanamycin (Kan; 50 μg/mL), gentamicin (Gm; 10 μg/mL), streptomycin (Sm; 50 μg/mL for *E. coli* and 60 μg/mL for *P. putida* ).

To study the growth parameters, most cultivations were performed in 48‐well microtiter plates. Precultures in 2.5 mL of LB were inoculated from frozen glycerol stocks in 3–4 replicates (as specified in figure legends). After 18 h at 30°C, 250 rpm (Unimax 1010, Heidolph), cultures were centrifuged (2000 g, 10 min, RT), washed and resuspended in 1 mL of M9 minimal salts medium. This medium contains per 1 L 4.25 g Na_2_HPO_4_·2H_2_O, 1.5 g KH_2_PO_4_, 0.25 g NaCl, 0.5 g NH_4_Cl, 2.5 mL of trace element solution (0.3 g/L H_3_BO_3_, 0.05 g/L ZnCl_2_, 0.03 g/L MnCl_2_·4H_2_O, 0.2 g/L CoCl_2_, 0.01 g/L CuCl_2_·2H_2_O, 0.02 g/L NiCl_2_·6H_2_O, 0.03 g/L (NH_4_)_2_MoO_4_·2H_2_O and 0.03 g/L FeSO_4_) (Abril et al. 1989) and MgSO_4_ of final concentration 2 mM. Cultivation was started by adding culture into a well containing 600 μL of minimal M9 medium supplemented with a carbon source (D‐glucose, D‐gluconate, citrate, D‐xylose; concentration specified in figure legends), with an initial optical density at 600 nm (OD_600_) of 0.05. Cultivations were carried out in Tecan Infinite M200 Pro, switching between circular and horizontal shaking while measuring the OD_600_ every 15 min. Growth at 30°C was monitored for at least 24 h.

In some cases (specified in figure legends), growth was also tested in shaken Erlenmeyer flasks. In this case, the preculture preparation conditions were identical to those for the 48‐well plates above. Fresh M9 medium with a carbon source of interest (concentration specified in figure legends) was inoculated with washed overnight culture to a starting OD_600_ of 0.1. The medium took up 1/10 of the flask volume to provide sufficient aeration. Cultures were then grown at 30°C, 200 rpm (IS‐971R shaker, Lab Companion or equivalent).

### 2.3 Calculation of Growth Parameters

Growth data were analysed using Microsoft Excel and OriginPro 2023 10.0.0.154 (OriginLab Corporation). Growth parameters were calculated by QurvE (Wirth et al. 2023) or the DeODorizer program (Swain et al. 2016) in the case of *E. coli* cultivation data. In some cases (flask experiments and plate cultivations with 0.1 g/L glucose), specific growth rate (μ) was determined manually during exponential growth as a slope of the data points obtained by plotting the natural logarithm of OD_600_ values against time. For biomass yield (*Y*_X/S_) calculations, biomass was determined as dry cell weight (DCW). Based on the previously prepared standard curve (Dvořák et al. 2024), one OD_600_ unit determined in *P. putida* EM42 culture in M9 medium is equivalent to 0.38 g L^−1^ of DCW. Biomass yield (*Y*_X/S_, where *X* represents biomass and *S* represents substrate) was calculated by plotting biomass concentration versus substrate concentration and quantifying the slope from regression analysis. Biomass‐specific substrate uptake rate (*q*_S_) and production rate (*q*_P_) were determined during exponential growth as described by Dvořák et al. (2024). Kinetic parameters *K*_S_ and *μ*_max_ were calculated by GraphPad Prism 10.3.0 (Dotmatics). For *E. coli* and *P. putida* EM42, the Monod kinetics Equation (1) was used as follows:

(1) $$\mu =\frac{\mu_{max}\left[S\right]}{K_{S}+\left[S\right]},$$

where *μ*_max_ is the maximum specific growth rate, *K*_S_ is the half‐saturation constant and [*S*] is the substrate concentration.

For *P. putida* EM42 ΔΔ (Table 1), the Monod substrate inhibition kinetics Equation (2) was used as follows:

(2) $$\begin{array}{c} \mu =\frac{\mu_{max}\left[S\right]}{K_{S}+[S]+\frac{{\left[S\right]}^{2}}{K_{i}}}, \end{array}$$

where *μ*_max_ is the maximum specific growth rate, if the substrate did not also inhibit cell growth, *K*_S_ is the half‐saturation constant describing the interaction of the cell and the substrate in the absence of inhibitor, [*S*] is the substrate concentration and *K*_i_ is the inhibition constant.

**TABLE 1:** Bacterial strains and plasmids used in this study.

Strain or plasmid	Characteristics	References
*Escherichia coli*		
CC118	Cloning host: *araD139* Δ*(ara‐leu)7697* Δ*lacX74 galE galK phoA20 thi−1 rpsE rpoB(RifR) argE(Am) recA1*	Manoil and Beckwith (1985)
*Pseudomonas putida*		
EM42	KT2440 derivative: Δprophages1,2,3,4 Δ*Tn7* Δ*endA1* Δ*endA2* Δ*hsdRMS* Δflagellum Δ*Tn4652*	Martínez‐García et al. (2014)
EM42 ΔΔ	EM42 with scarless deletion of the *gcd* gene (*PP_1444*) encoding periplasmic glucose dehydrogenase and the *hexR* gene (*PP_1021*) encoding DNA‐binding transcriptional regulator	Dvořák et al. (2024)
EM42 Δ*oprB*	EM42 with premature stop codons in three *oprB* genes, *PP_1019, PP_1445* and *PP_3570*	This study
EM42 ΔCPG	EM42 with premature stop codons in three Candidate Porin Genes (CPG), *PP_0504, PP_2662* and *PP_2702*	This study
EM42 ΔΔ Δ*oprB*	EM42 Δ*gcd* Δ*hexR* with premature stop codons in three *oprB* genes, *PP_1019, PP_1445* and *PP_3570*	This study
EM42 ΔΔ ΔCPG	EM42 Δ*gcd* Δ*hexR* with premature stop codons in three porin genes, *PP_0504, PP_2662* and *PP_2702*	This study
EM42 XYL^a^	EM42 bearing the pSEVA2213_*xylABE* L3 plasmid	This study
EM42 ΔΔ XYL	EM42 ΔΔ bearing the pSEVA2213_*xylABE* L3 plasmid	This study
EM42 Δ*oprB* XYL	EM42 Δ*oprB* bearing the pSEVA2213_*xylABE* L3 plasmid	This study
EM42 ΔΔ Δ*oprB* XYL	EM42 ΔΔ Δ*oprB* bearing the pSEVA2213_*xylABE* L3 plasmid	This study
EM42 ΔCPG XYL	EM42 ΔCPG bearing the pSEVA2213_*xylABE* L3 plasmid	This study
EM42 ΔΔ ΔCPG XYL	EM42 ΔΔ ΔCPG bearing the pSEVA2213_*xylABE* L3 plasmid	This study
EM42 XYL *oprB*‐I	EM42 bearing the pSEVA2213_*xylABE*_*oprB*‐I plasmid	This study
EM42 ΔΔ XYL *oprB*‐I	EM42 ΔΔ bearing the pSEVA2213_*xylABE_oprB*‐I plasmid	This study
EM42 Δ*oprB* XYL *oprB*‐I	EM42 Δ*oprB* bearing the pSEVA2213_*xylABE_oprB*‐I plasmid	This study
EM42 ΔΔ Δ*oprB* XYL *oprB*‐I	EM42 ΔΔ Δ*oprB* bearing the pSEVA2213_*xylABE_oprB*‐I plasmid	This study
*Plasmids*		
pEX‐A128_ gRNAtemplate	Delivery vector used by Eurofins Genomics for synthesized constructs here containing gRNA and *csy4* responsible for sgRNA processing, Amp^R^	Volke et al. (2022)
pBEC_pnCas9‐6‐UGI‐Csy4::Ptrc	Base editor plasmid, Gm^R^	Volke et al. (2022)
pBEC_*oprB*	Base editor with 6 spacers targeting *PP_1019*, *PP_1445* and *PP_3570* (two spacers per gene)	This study
pBEC_CPG	Base editor with 6 spacers targeting *PP_0504*, *PP_2662* and *PP_2702* (two spacers per gene)	This study
pSEVA2213	Expression vector: *oriV(RK2) pEM7 neo*, Km^R^	Martinez‐Garcia et al. (2022)
pSEVA2213_*xylABE* L3	Vector pSEVA2213 with synthetic *xylABE* operon encoding XylA xylose isomerase, XylB xylulokinase, XylE xylose‐proton symporter from *Escherichia coli* with putative transcriptional coupler upstream of *xylA* which arose during adaptive laboratory evolution, isolated from strain PD855 in Dvořák et al. 2024	Dvořák et al. (2024)
pSEVA2213_*xylABE*_*oprB*‐I L3	Vector pSEVA2213_*xylABE* L3 with a synthetic ribosome‐binding site and a native porin gene *oprB‐I* from *P. putida*	This study
pSEVA438_*tal*	Expression vector: pBBR1 *xylS*‐Pm, Sm^R^/Sp^R^ with transaldolase gene *tal* from *P. putida* (*PP_2168*)	Dvořák et al. (2024)

^a^ All XYL strains were also transformed with the pSEVA438_*tal* plasmid when grown on xylose or mixture of glucose and xylose. This plasmid was not present when the XYL strains were grown only on glucose.

Statistical significance, where appropriate, was calculated using the unpaired Welch's *t*‐test calculated by the GraphPad *t*‐Test Calculator (Equation (3) https://www.graphpad.com/quickcalcs/ttest1/)

(3) $$\begin{array}{c} t=\frac{\bar{x_{1}}-\bar{x_{2}}}{\sqrt{\left(\frac{s_{1}^{2}}{n_{1}}+\frac{s_{2}^{2}}{n_{2}}\right)}}, \end{array}$$

where *x* is the mean value, *s* is the standard deviation, *n* is the number of replicates, and (1) and (2) denote each of the compared groups.

### 2.4 Strain Construction

For PCR amplifications, Q5 High‐Fidelity DNA Polymerase (New England Biolabs, NEB) or Phusion High‐Fidelity DNA Polymerase (Thermo Fisher Scientific) was routinely used according to the manufacturer's instructions. Amplicons were gel‐purified using NucleoSpin Gel and PCR Clean‐up (Macherey‐Nagel, or equivalent) and then incorporated into a suitable vector using T4 DNA ligase (NEB). Chemocompetent *E. coli* cells CC118 were transformed with the ligation mixture according to the manufacturer's instructions (Zymo Research). Single colonies were selected on LB agar plates containing the corresponding antibiotics and then tested by colony PCR using DreamTaq Green PCR Master Mix 2× (Thermo Fisher Scientific). Base editing was performed according to the protocol published by Volke et al. (2022). Two different spacers targeted each gene of interest. Suitable positions for base editing were identified using CRISPy‐web (https://crispy.secondarymetabolites.org), and spacer sequences were generated by a tool published in the above‐mentioned study (Volke et al. 2022) (Table S1 in Supporting Information). Positions of premature STOP codons introduced by the base editing system in selected porin genes are summarized in Table S2. Two plasmids were prepared; first targeting all three *oprB* genes (pBEC_*oprB*) and second targeting the three other Candidate Porin Genes (pBEC_CPG). Plasmids pBEC_*oprB*, pBEC_CPG, pSEVA2213_*xylABE* L3 (Dvořák et al. 2024), pSEVA2213_*xylABE*_*oprB*‐I L3 (Sequence S1 in Supporting Information) and pSEVA438_*tal* (Table 1) were introduced to the strains of interest by electroporation. Electrocompetent cells were prepared by washing the biomass several times in 0.3 M sucrose solution (Choi et al. 2006). All electroporations were performed using GenePulser Xcell (Biorad, 2500 V, 25 μF, 200 Ω, 2 mm). Strains transformed with pBEC plasmids were recovered in liquid LB with gentamicin, and after plating on an agar plate with 5% (w/v) sucrose, single colonies were tested for the loss of the plasmid by selection for susceptibility to gentamicin. Genome editing was tested by colony PCR and sequencing (SEQme, Czech Republic). Transformants with pSEVA plasmids were selected from an LB agar plate with kanamycin or streptomycin. Single colonies were re‐streaked and stored as glycerol stocks at −60°C. All strains and plasmids used in this study are listed in Table 1.

Strains prepared for the growth on xylose or mixture of glucose and xylose carry two plasmids: (1) pSEVA2213_*xylABE* L3 (or pSEVA2213_*xylABE*_*oprB*‐I L3) encoding xylose isomerase pathway from *E. coli* (XylA, XylB and XylE xylose transporter and bearing putative transcriptional coupler upstream of *xylA*, Tables 1 and 2) pSEVA438_*tal*, bearing *P. putida* innate Tal transaldolase gene, whose overexpression was previously shown to improve growth on xylose (Dvořák et al. 2024). These strains and other strains bearing the pSEVA2213 plasmid with *xyl* genes (including those used for growth on glucose) are denoted XYL for clarity (e.g., EM42 XYL and EM42 ΔΔ XYL *oprB‐*I). The pSEVA438_*tal* plasmid was not present in the XYL strains grown solely on glucose. All primers used in this study are listed in Table S3.

### 2.5 Whole Genome Sequencing of Porin Mutants

For Illumina sequencing, the genomic DNA of EM42 Δ*oprB* and EM42 ΔΔ Δ*oprB* was isolated using the RTP Bacteria DNA Mini Kit (STRATEC Molecular) following the manufacturer's instructions. Sequencing was performed by Plasmidsaurus (https://www.plasmidsaurus.com), with custom analysis and annotation.

For in‐house Oxford Nanopore Technologies (ONT) sequencing, genomic DNA of the strains was extracted using the Genomic DNA Clean & Concentrator‐25 kit (Zymo Research) according to the manufacturer's instructions. Cells in mid‐exponential phase, grown in LB at 30°C, lysed with NEBExpress T4 lysozyme (NEB) in 10 mM Tris–HCl buffer (pH 7.5) with the addition of RNase A (NEB) and proteinase K (Sigma), were used for DNA isolation (Dvořák et al. 2024). Sequencing libraries were prepared using a Ligation Sequencing Kit V14 (SQK‐LSK114) and sequenced with FLO‐FLG114 flow cells (R10.4.1) in a MinION device (ONT). The device was controlled by the software MinKNOW version 25.09.16 (ONT), which was also used for super‐accurate base calling with a minimum Q score of 10 and trimming.

The complete genome sequences were assembled using Flye v2.9.6 with 2 polishing iterations (Lin et al. 2016), followed by manual inspection in homopolymer and ambiguous sites using Illumina reads mapped by Bowtie2 v2.4.5 plugin (‘End to end’ alignment type and ‘Medium sensitivity’ preset) in Geneious Prime 2026.0.2 (https://www.geneious.com). Whole‐genome sequences were annotated by the NCBI Prokaryotic Genome Annotation Pipeline (Li et al. 2020) and compared with the reference sequence of strain EM42 Δ*gcd* Δ*hexR* pSEVA2213_*xylABE* (strain PD584) (Dvořák et al. 2024) using the Mauve Plugin (Darling et al. 2010) in Geneious Prime 2026.0.2. Changes affecting encoded protein sequences were found and analysed (Data S1).

### 2.6 Analytical Methods

The optical density in cell cultures was recorded at 600 nm using a UV/VIS spectrophotometer Genesys 180 (Thermo Fisher Scientific). Samples from cultures in shaken flasks were collected by withdrawing 0.5 mL of culture medium. The sample was then centrifuged (20,000 g, 10 min). The supernatant was filtered through 4 mm/0.45 μm LUT Syringe Filters (Labstore) and stored at −20°C. Prior to the HPLC analysis, 50 mM H_2_SO_4_ was added to the samples at a 1:1 ratio to stop any hydrolytic activity and to dilute the samples. High‐performance liquid chromatography (HPLC) was used to quantify glucose, xylose, gluconate, 2‐ketogluconate and xylonate. HPLC analysis was carried out using an Agilent 1100 Series system (Agilent Technologies) equipped with a refractive index detector and a Hi‐Plex H, 7.7 × 300 mm, 8 μm HPLC column (Agilent Technologies) and a variable wavelength detector (Agilent Technologies). Analyses were performed using the following conditions: mobile phase 5 mM H_2_SO_4_, mobile phase flow 0.5 mL/min, injection volume 20 μL, column temperature 65°C, RI detector temperature 55°C. Standards of analysed carbohydrates (Sigma‐Aldrich) were used for the preparation of calibration curves.

### 2.7 Statistical Analyses

The number of repeated experiments or biological replicates is specified in figure legends. The mean values and corresponding standard deviations are presented. When appropriate, data were treated with a two‐tailed Student's *t*‐test and confidence intervals were calculated for the given parameters to test a statistically significant difference in means between two experimental datasets.

## 3 Results

### 3.1 Selection of Candidate Porin Genes

To determine which protein channels are involved in glucose uptake in *P. putida* , a list of candidate porin‐encoding genes was prepared comprising 38 genes in total (Table 2). To narrow the list down, the genes were further characterized *in silico* using a range of publicly available comparison and prediction tools (BLAST, Swiss model, Rosetta, AlphaFold Server) and previously published experimental evidence. Most of the candidate genes (23 out of 38) were identified as members of the *oprD* family comprising amino acid‐specific porins. One gene, *pcaP (PP_1382)*, has been annotated as a porin even though it is almost identical (99% on the amino acid level) to 2‐hydroxy fatty acid dioxygenase (Seki et al. 2019). Four genes fall into the *ompA* family. Generally, these monomeric porins consist of a beta‐barrel and a periplasmic domain interacting with the peptidoglycan layer. Their functions range from facilitating the diffusion of substrates to stabilizing the membrane structure and interaction with the outer environment (Chen et al. 2015; Zhou et al. 2023). Interestingly, in *P. putida* , a portion of the barrel‐forming β‐strands is replaced with α‐helices (compare e.g., UniProt structures P0A910 for *E. coli* OmpA protein and Q88DZ9 for *P. putida* PP_4669). To the best of our knowledge, no transport function has been associated with any of these proteins; however, analyses reveal that they are all lipoproteins (SignalP—6.0, Nielsen 2025) and could therefore have structural and/or immunogenic function. Comparison with porins in other bacteria (*E. coli, Klebsiella pneumoniae* and *Xanthomonas oryzae* ) showed that non‐pore‐forming porins are relatively uncommon (Table S4). Lastly, OprF (*PP_2089*) was not further analysed as it has a structural function ensuring the stability of the outer membrane, and its downregulation compromises cell fitness and boosts membrane vesicle formation (Wilkes et al. 2025; Chevalier et al. 2017). Other genes encode aquaporins (*PP_1076* and *PP_4282*), phosphate‐selective porin *oprP* (*PP_0037*), and a ferric citrate porin (*PP_4613*). Ultimately, six genes were selected for experimental analysis (Table 2). Three porins (*PP_1019*, *PP_1445* and *PP_3570*) fall into the OprB family, which comprises carbohydrate‐selective porins. Three other selected poorly characterized genes (*PP_0504*, *PP_2662* and *PP_2702*) have unique sequences within the *P. putida* KT2440 genome, and structure predictions revealed they could form a pore. To the best of our knowledge, no record of transport function is available for any of these proteins.

**TABLE 2:** Annotated porins of *Pseudomonas putida* KT2440.

LocusTag	Gene name	Product description	Porin	Protein family
***PP_1019***	*oprB*‐I	Carbohydrate‐selective porin	Yes	OprB
***PP_1445***	*oprB*‐II	Carbohydrate‐selective porin	Yes	
***PP_3570***	*oprB*‐III	Carbohydrate‐selective porin	Yes	
***PP_0504***	*oprG*	Outer membrane protein OprG	Yes	—
***PP_2662***	—	Hypothetical protein	Yes	—
***PP_2702***	—	Porin	Yes	—
*PP_0046*	*opdT*‐I	Tyrosine‐specific outer membrane porin D	Yes	OprD
*PP_0234*	*oprE*	Outer membrane porin E	Yes	
*PP_0268*	*oprQ*	Outer membrane porin D	Yes	
*PP_0799*	*opdC*	Histidine‐specific outer membrane porin D	Yes	
*PP_0883*	*opdP*	Glycine‐glutamate dipeptide porin	Yes	
*PP_1173*	*galP*‐I	Porin‐like protein	Yes	
*PP_1206*	*oprD*	Basic amino acid specific porin OprD	Yes	
*PP_1383*	*galP*‐II*/BenF*‐like porin	Porin‐like protein	Yes	
*PP_1419*	*opdH*	Tricarboxylate‐specific outer membrane porin	Yes	
*PP_2058*	—	Porin	Yes	
*PP_2089*	*oprF*	Porin F	Yes	
*PP_2517*	*gllP*	Porin‐like protein	Yes	
*PP_2754*	—	OprD family outer membrane porin	Yes	
*PP_3168*	*nicP*‐I	Porin‐like protein	Yes	
*PP_3271*	*phaK*	Phenylacetic acid‐specific porin	Yes	
*PP_3390*	—	Porin	Yes	
*PP_3630*	*opdT*‐II	Tyrosine‐specific outer membrane porin D	Yes	
*PP_3656*	—	Aromatic compound‐specific porin	Yes	
*PP_3739*	*galP*‐IV	Porin‐like protein	Yes	
*PP_3764*	*opdN*	Outer‐membrane porin D	Yes	
*PP_3939*	*nicP*‐II	Porin‐like protein	Yes	
*PP_4465*	—	Porin	Yes	
*PP_5250*	*opdB*	Proline‐specific outer membrane porin D	Yes	
*PP_0773*	—	OmpA/MotB domain‐containing protein	No	OmpA
*PP_1122*	—	OmpA family protein	No	
*PP_1128*	—	OmpA family protein	No	
*PP_4669*	—	OmpA family protein	No	
*PP_0037*	*oprP*	Porin P	Yes	—
*PP_1076*	*glpF*	Aquaglyceroporin	Yes	—
*PP_1382*	*pcaP*	Porin	No	—
*PP_4282*	*aqpZ*	Aquaporin Z	Yes	—
*PP_4613*	*fecA*	Outer membrane ferric citrate porin	Yes	—

*Note:* Genes chosen for the experimental part of this study are written in bold. The ability of the protein to form a pore was determined based on published or predicted tertiary structures.

Selected genes were knocked out using the cytidine base editor tailored for Gram‐negative bacteria (Volke et al. 2022) using two plasmid constructs, pBEC_*oprB* (for *oprB‐*I, *oprB‐*II and *oprB‐*III) and pBEC_CPG (for *PP_0504*, *PP_2662* and *PP_2702*) (see Section 2). Point mutations resulting in premature STOP codons were introduced in *P. putida* EM42 and EM42 ΔΔ (Table S2). The resulting mutant strains were named EM42 Δ*oprB*, EM42 ΔCPG, EM42 ΔΔ Δ*oprB* and EM42 ΔΔ ΔCPG (for simplicity, the Δ symbol is used for functional knockout as well as deletion of an entire gene sequence; ΔΔ denotes Δ*gcd* Δ*hexR* in this manuscript). The desired mutations were confirmed in all mutant strains by Sanger sequencing of the locus (SEQme, Czech Republic). Additionally, we sequenced the whole genome of Δ*oprB* mutants using Illumina and ONT. Among the observed mutations that can be considered off‐target edits by the base editor (Data S1; Volke et al. 2022), we identified one in the *pgi‐1* gene (*PP_1808*) of the EM42 ΔΔ Δ*oprB* strain. Although this mutation leads to the premature termination (truncation) of the glycolytic isoenzyme glucose‐6‐phosphate isomerase 1—resulting in a polypeptide roughly one‐third shorter than the wild type—we did not expect it to significantly affect the growth of *P. putida* on sugars. Previous studies have shown that even the simultaneous deletion of both *pgi‐1* and *pgi‐2* does not markedly reduce growth on glucose (Nikel et al. 2015; Bentley et al. 2020; Dvořák et al. 2024). Furthermore, while the double deletion prevents the growth of recombinant *P. putida* on xylose, this effect is primarily attributed to the loss of *pgi‐2* (Ling et al. 2022; Dvořák et al. 2024).

### 3.2 OprB Porins Are Important for Glucose Uptake

**FIGURE 2:**
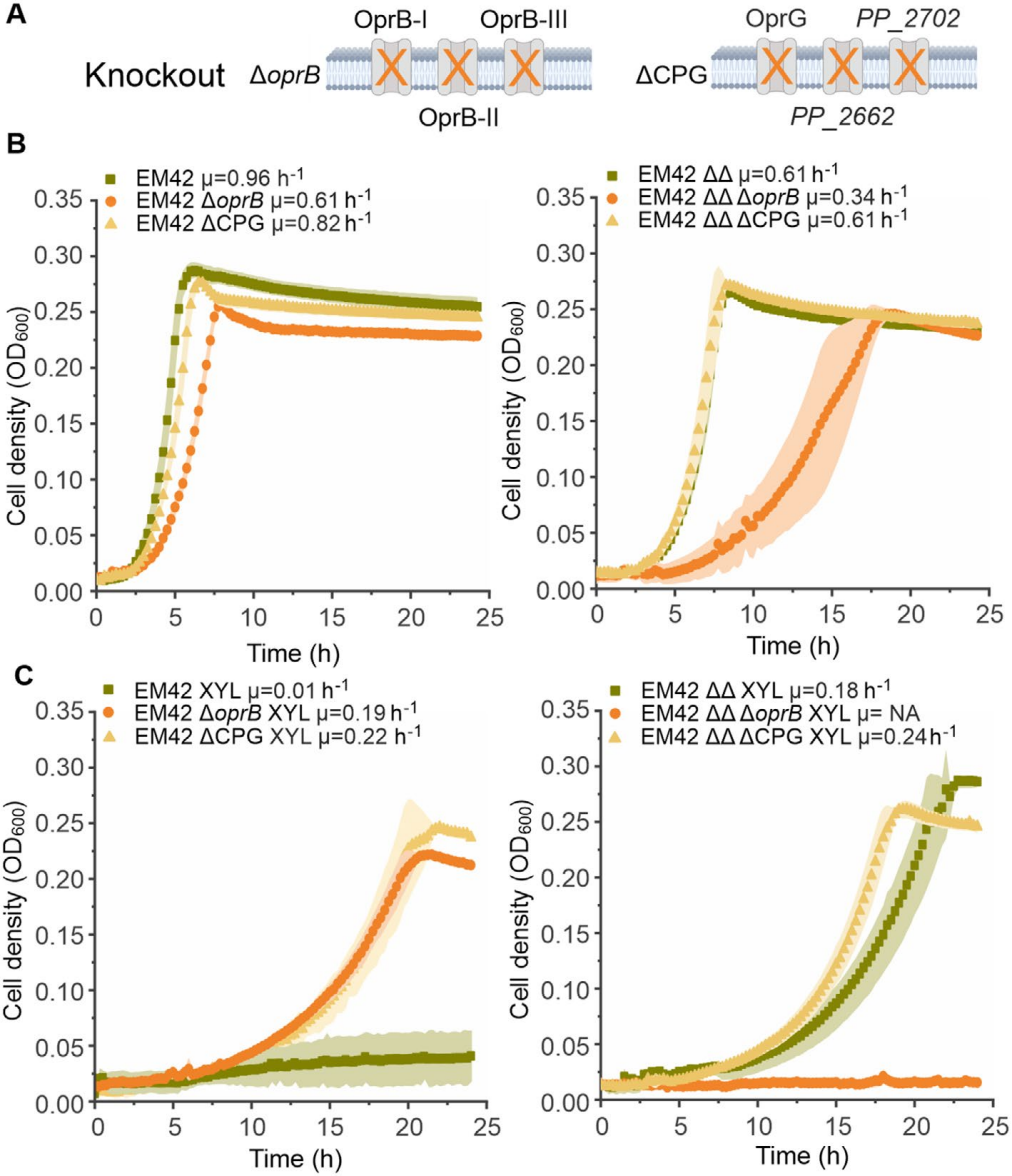
The effect of inactivation of selected groups of porins in two *Pseudomonas putida* strains on their growth on glucose and xylose. (A) Schematic representation of knocked out groups of porins and the absence of these porins in the outer phospholipid bilayer (the individual components in the scheme are not to scale). Cultivation in minimal M9 medium supplemented with 1 g/L of glucose (B) or xylose (C) was conducted in a 48‐well microtiter plate. Glucose‐growing strains are supplemented with Kan, and xylose‐growing strains are supplemented with Kan, Sm and 3‐methylbenzoate (25 μM). Abbreviations: ΔCPG, knockout of *PP_0504*, *PP_2662* and *PP_2702*; Δ*oprB*, knockout of *oprB*‐I, *oprB*‐II and *oprB*‐III. Shown are means ± standard deviations from four (*n* = 4) biological replicates. Scheme (A) was created by BioRender.com.

First, we tested the effect of the inactivation of selected porin genes on the growth on D‐glucose as a sole carbon source (Figure 2A,B and Figure S1A–C in Supporting Information). For *P. putida* , sugars are not preferred substrates. In the rhizosphere environment, they are only present in very low concentrations (Lugtenberg et al. 1999), so we presumed the uptake mechanisms would reflect this. For that reason, we grew *P. putida* mutant strains on a range of concentrations of glucose (0.1, 0.25, 0.5, 1.0, 2.0 and 10 g/L; Figure 2B, Figures S1 and S2).

The inactivation of OprB porins had a negative effect on the growth on glucose in both parental strains, more so in the engineered EM42 ΔΔ (Figure 2B, Figures S1 and S2, Table S5). The knockout of the second group of porins, CPG, had little (EM42) or no impact (EM42 ΔΔ) across different glucose concentrations. For that reason, these mutants were not investigated further in the following experiments with glucose, and we focused on OprB porins. The tested strains grew the fastest with 1 g/L glucose (Figure S2 and Table S5), and therefore this concentration was adopted for the following cultivations in microtiter plates. With higher substrate concentrations, the growth rate either stagnated (EM42 and EM42 Δ*oprB* strains) or declined (EM42 ΔΔ and EM42 ΔΔ Δ*oprB*). At the lowest glucose concentration tested (0.1 g/L), *oprB* inactivation had a very pronounced effect not only in the EM42 ΔΔ but also in the EM42 strain (Figure S1 and Table S6). This suggests that glucose passage through the outer membrane is not a limiting factor for sugar utilization in its higher concentrations, unlike downstream steps.

Having growth data across various glucose concentrations also allowed us to analyse microbial growth kinetics using models analogous to enzyme kinetics. Specifically, we fit the data to Monod‐type equations, estimating *K*_S_ (half‐saturation constant) and *μ*_max_ (maximum specific growth rate) for each strain (Figure S2A–C). The obtained *K*_S_ values are similar for *E. coli* , a Gram‐negative bacterial model, and *P. putida* EM42 and EM42 ΔΔ (0.04 mM, 0.08 mM, and 0.06 mM, respectively), but *oprB* knockout results in an increase in *K*_S_ values (0.13 mM and 0.49 mM for EM42 Δ*oprB* and EM42 ΔΔ Δ*oprB*, respectively) (Table S7), suggesting lower apparent substrate affinity.

### 3.3 OprB Porins Are Involved in the Transport of Xylose and Some Other Substrates

D‐Xylose, another sugar prevalent in lignocellulose, is also an important substrate for the next‐generation biotechnologies (Jagtap and Rao 2018; Narisetty et al. 2021). Xylose is not naturally metabolized by *P. putida* ; however, as recently shown by several research teams, fast growth of the bacterium on this pentose and xylose valorization can be achieved through metabolic engineering strategies (Dvořák et al. 2024; Bator et al. 2020). Here, we wanted to investigate the extent to which transport of xylose across the outer membrane and into the periplasm influences the utilization of this non‐native substrate. All strains tested for growth on xylose (marked with the abbreviation XYL) were additionally transformed with pSEVA2213_*xylABE* L3 carrying *xylABE* genes from *E. coli* and pSEVA438_*tal* plasmid with cloned innate transaldolase gene from *P. putida* (Table 1) (Dvořák et al. 2024). OprB porins are crucial for xylose uptake in EM42 ΔΔ XYL, as the strain with knocked‐out porins does not grow on xylose (Figure 2C and Table S5). Interestingly, the inactivation of the *oprB* genes and candidate porin genes (CPG) proved advantageous in EM42 (Figure 2C).

We argue that the positive effect of the knockouts in the background of EM42 strain is rather indirect. In a recent study from our laboratory, we performed proteomic analysis of several engineered *P. putida* strains grown on xylose (Supporting Information 2 in Dvořák et al. 2024 and PRIDE dataset identifier PXD047537). Here, we re‐examined this dataset and found that OprB‐I (UniProt ID: Q88P34) and the CPG‐set porins OprG (UniProt ID: Q88QI8) and PP_2662 (UniProt ID: Q88JI7) were detected in all xylose‐grown samples at relatively high abundance, displaying a high basal level of expression even in the absence of *P. putida* natural sugar inductor glucose. Based on protein abundance estimates (median DIA‐TPA values from 4 replicates, He et al. 2019), OprB‐I ranked among the top 5% of detected proteins, whereas OprG ranked among the top 35% and PP_2662 among the top 40%. The other OprB and CPG porins showed lower or no abundance: OprB‐III (UniProt ID: Q88GZ5) ranked among the bottom 25% of the least abundant proteins and the candidate CPG porin PP_2702 (UniProt ID: Q88JE7) and OprB‐II (UniProt ID: Q88MX3) were not detected using this proteomic method. Although obtained in *P. putida* strains different from those used in this study, these results support the possibility that knockout of the abundant OprB‐I, OprG and PP_2662 porins could free secretion capacity for other proteins, such as heterologously expressed xylose transporter XylE. We conclude that CPG porins do not play a key role in the transport of glucose and xylose in the tested *P. putida* strains and focus solely on OprB porins in subsequent work.

Mutants with inactivated OprB porins were also grown on gluconate (a product of glucose oxidation in *P. putida* periplasm, Figures 1 and S3B) and gluconeogenic substrate citrate (Figure S3A). In both cases, inactivation of OprB porins resulted in a lower growth rate (Table S5). The negative effect of OprB inactivation was more pronounced in the EM42 ΔΔ strain. These experiments indicate that OprB porins also play a role in the transport of non‐sugar substrates gluconate and citrate.

### 3.4 OprB‐I Alone Functionally Complements All OprB Porins

**FIGURE 3:**
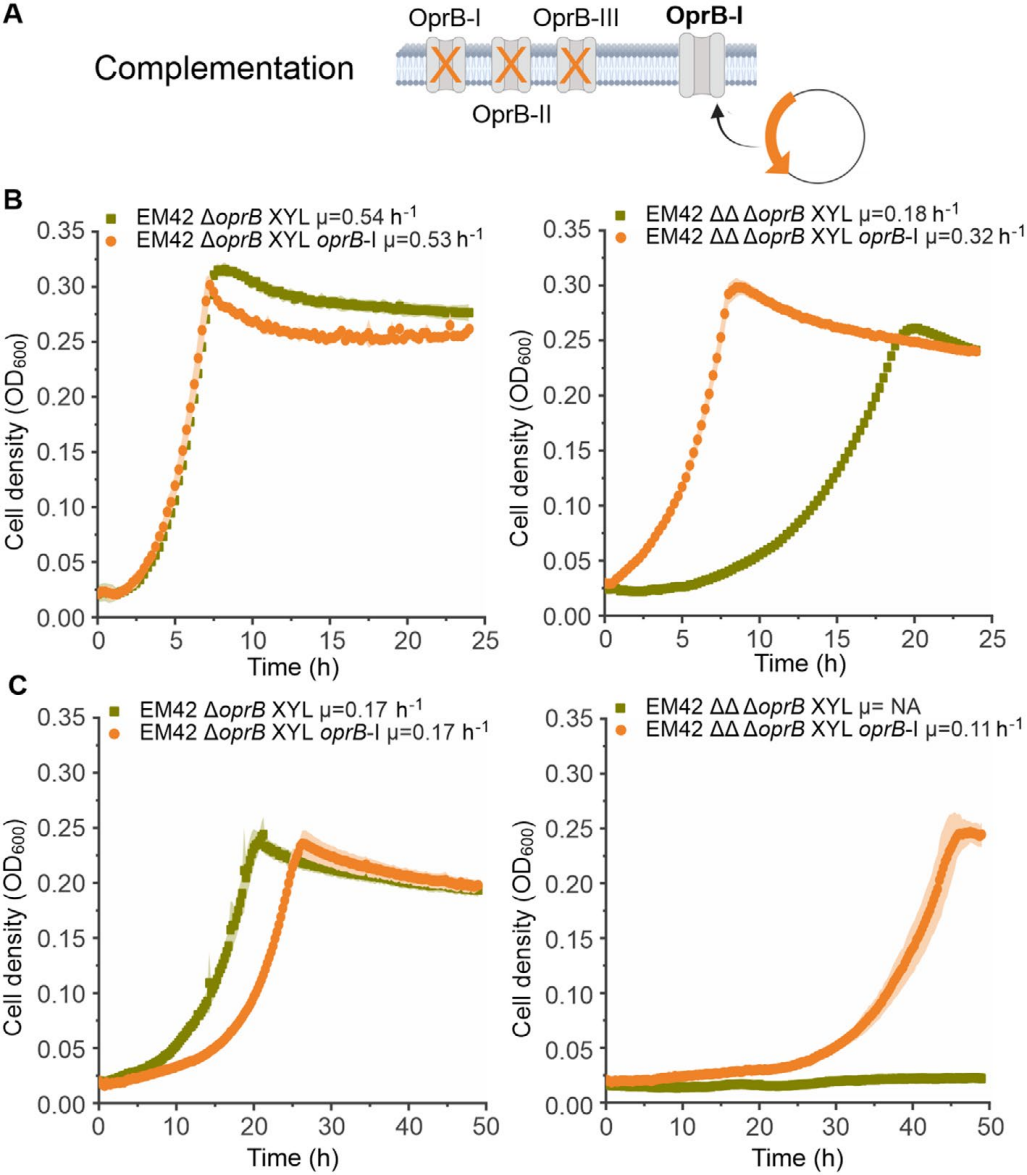
The effect of complementation of OprB‐I in engineered *Pseudomonas putida* strains grown on glucose or xylose. (A) Schematic representation of functional complementation via the OprB‐I porin in the outer phospholipid bilayer (the individual components in the scheme are not to scale). Cultivation on minimal M9 medium supplemented with 1 g/L glucose (B) or xylose (C) was conducted in a 48‐well MTP. Glucose‐growing strains are supplemented with Kan, and xylose‐growing strains are supplemented with Kan, Sm and 3‐methylbenzoate (25 μM). Shown are means ± standard deviations from at least three (*n* = 3) biological replicates. Scheme (A) was created by BioRender.com.

To confirm the role of OprB in the *P. putida* metabolism of two major lignocellulosic sugars, glucose and xylose, *oprB*‐I (*PP_1019*) was overexpressed in both tested strains with knocked‐out *oprB* genes (Figure 3A). Our goal was to test the effect of OprB porins on the co‐utilization of glucose and xylose by single strains. To this end, we placed the *oprB‐*I gene downstream of the *xyl* synthetic operon in the plasmid pSEVA2213_*xylABE* L3 (Table 1) and transformed EM42 Δ*oprB* and EM42 ΔΔ Δ*oprB* with the resulting construct pSEVA2213_*xylABE*‐*oprB‐*I L3. This gave rise to strains designated EM42 Δ*oprB* XYL *oprB‐*I and EM42 ΔΔ Δ*oprB* XYL *oprB‐*I. Respective strains transformed with pSEVA2213_*xylABE* L3 without the *oprB*‐I gene (EM42 Δ*oprB* XYL and EM42 ΔΔ Δ*oprB* XYL) were used as controls. All strains tested for growth on xylose were additionally transformed with the pSEVA438_*tal* plasmid (Dvořák et al. 2024). The *oprB*‐I complementation had a significantly positive effect (*p* < 0.05) on the growth of EM42 ΔΔ Δ*oprB* strain on both substrates (Figure 3B,C, Table S5). In this case, the OprB‐I porin alone was probably enough to facilitate the necessary substrate transport into the periplasm. On the other hand, in the EM42 Δ*oprB* strain, complementation with *oprB*‐I had a neutral or slightly negative effect (Figure 3B,C, Table S5).

### 3.5 OprB‐I Overexpression Improves the Growth on Glucose and Xylose

**FIGURE 4:**
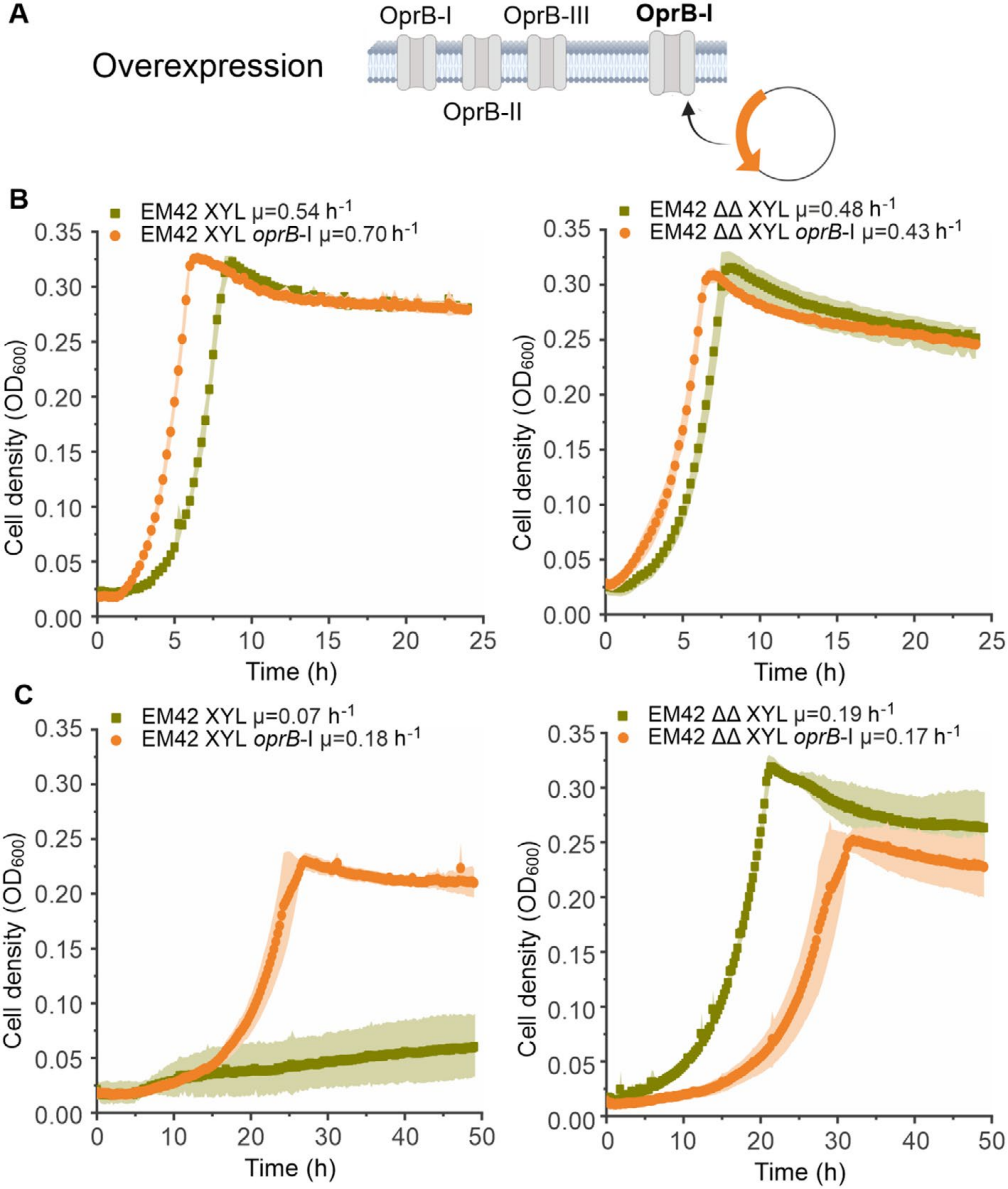
The effect of overexpression of *oprB‐*I in engineered *Pseudomonas putida* strains grown on glucose or xylose. (A) Schematic representation of the *oprB*‐I overexpression and its increased amount in the outer phospholipid bilayer (the individual components in the scheme are not to scale). Cultivation on minimal M9 medium supplemented with 1 g/L glucose (B) or xylose (C) was conducted in a 48‐well MTP. Glucose‐growing strains are supplemented with Kan, and xylose‐growing strains are supplemented with Kan, Sm and 3‐methylbenzoate (25 μM). Shown are means ± standard deviations from at least three (*n* = 3) biological replicates. Scheme (A) was created by BioRender.com.

We also tested overexpression of *oprB‐*I in strains with intact OprB porins (Figure 4A). EM42 and EM42 ΔΔ strains were transformed with pSEVA2213_*xylABE*‐*oprB‐*I L3 or pSEVA2213_*xylABE* L3 (controls) to give rise to EM42 XYL *oprB*‐I and EM42 ΔΔ XYL *oprB*‐I or EM42 XYL and EM42 ΔΔ XYL, respectively. For growth on xylose, strains were additionally transformed with the pSEVA438_*tal* plasmid. Intriguingly, *oprB*‐I overexpression alone increased the growth rate in the EM42 strains on both sugars, glucose and xylose, significantly (*p* < 0.05, Figure 4B,C, Table S5). However, in the EM42 ΔΔ XYL strain previously optimized for growth on xylose (Dvořák et al. 2024), the growth rate on this sugar was significantly (*p* < 0.05) reduced when *oprB*‐I was overexpressed (Figure 4C and Table S5).

In all cases, the plasmid carrying the *oprB‐*I gene for complementation or overexpression also encoded the synthetic *xylABE* operon. Because the *xylE* transporter was reported to bind and likely transport glucose in addition to xylose (Sun et al. 2012), we tested the effect of *oprB*‐I overexpression on glucose in shaken flasks (Figure S4A–C). Comparison with controls with either empty pSEVA2213 vector, the same plasmid containing the sole *xylABE* synthetic operon and the vector carrying *xylABE* synthetic operon and additionally *oprB*‐I proved that *oprB‐*I overexpression is advantageous for glucose utilization (*μ* = 0.51 ± 0.00 h^−1^ for EM42 XYL *oprB*‐I vs. 0.42 ± 0.01 h^−1^ for EM42 XYL). Increased growth rate was accompanied by decreased levels of secreted gluconate and 2‐ketogluconate, as previously reported (Volke et al. 2023), decreased specific gluconate production rate (*q*_P_ = 5.09 ± 0.08 mmol_GLN_/g_DCW_/h for EM42 XYL *oprB*‐I vs. 15.33 ± 0.75 mmol_GLN_/g_DCW_/h for EM42 XYL), and increased biomass yield (*Y*_X/S_ = 0.21 ± 0.01 g_DCW_/g_glucose_ for EM42 XYL *oprB*‐I vs. 0.15 ± 0.02 g_DCW_/g_glucose_ for EM42 XYL). EM42 XYL *oprB*‐I consumed all substrate in 6 h (Figure S4C), compared to 8 h it took the control strain, EM42 XYL (Figure S4B). This shows that the *oprB*‐I overexpressing strain transforms glucose into biomass more efficiently than EM42 XYL control.

As shown in previous sections, both *oprB* inactivation and *oprB*‐I overexpression had a positive effect on the growth of EM42 XYL on xylose. To better understand the underlying processes, we cultivated these strains in flasks, which enabled us to measure xylose and xylonate concentrations throughout the cultivation. Cultivation experiments confirmed that both the *oprB‐I* knockout and overexpression substantially accelerate the growth of *P. putida* on xylose (Figure S4D–F). The growth profiles indicate that this is due to slower xylose oxidation during the initial 15 h of EM42 Δ*oprB* XYL (*q*_P_ = 1.85 ± 0.07 mmol_XLN_/g_DCW_/h) and EM42 XYL *oprB*‐I (*q*_P_ = 2.29 ± 0.27 mmol_XLN_/g_DCW_/h) cultures compared to EM42 XYL cultures (*q*_P_ = 5.70 ± 0.19 mmol_XLN_/g_DCW_/h). At the same time, xylose was utilized more efficiently by EM42 Δ*oprB* XYL (*Y*_X/S_ = 0.21 ± 0.02 g_DCW_/g_xylose_) and EM42 XYL *oprB‐*I (*Y*_X/S_ = 0.13 ± 0.04 g_DCW_/g_xylose_) strains than by EM42 XYL (*Y*_X/S_ = 0.04 ± 0.01 g_DCW_/g_xylose_).

### 3.6 Co‐Utilization Experiments in Flasks Confirm the Positive Effect of oprB‐I Overexpression on the Dynamics of Glucose and Xylose Uptake

**FIGURE 5:**
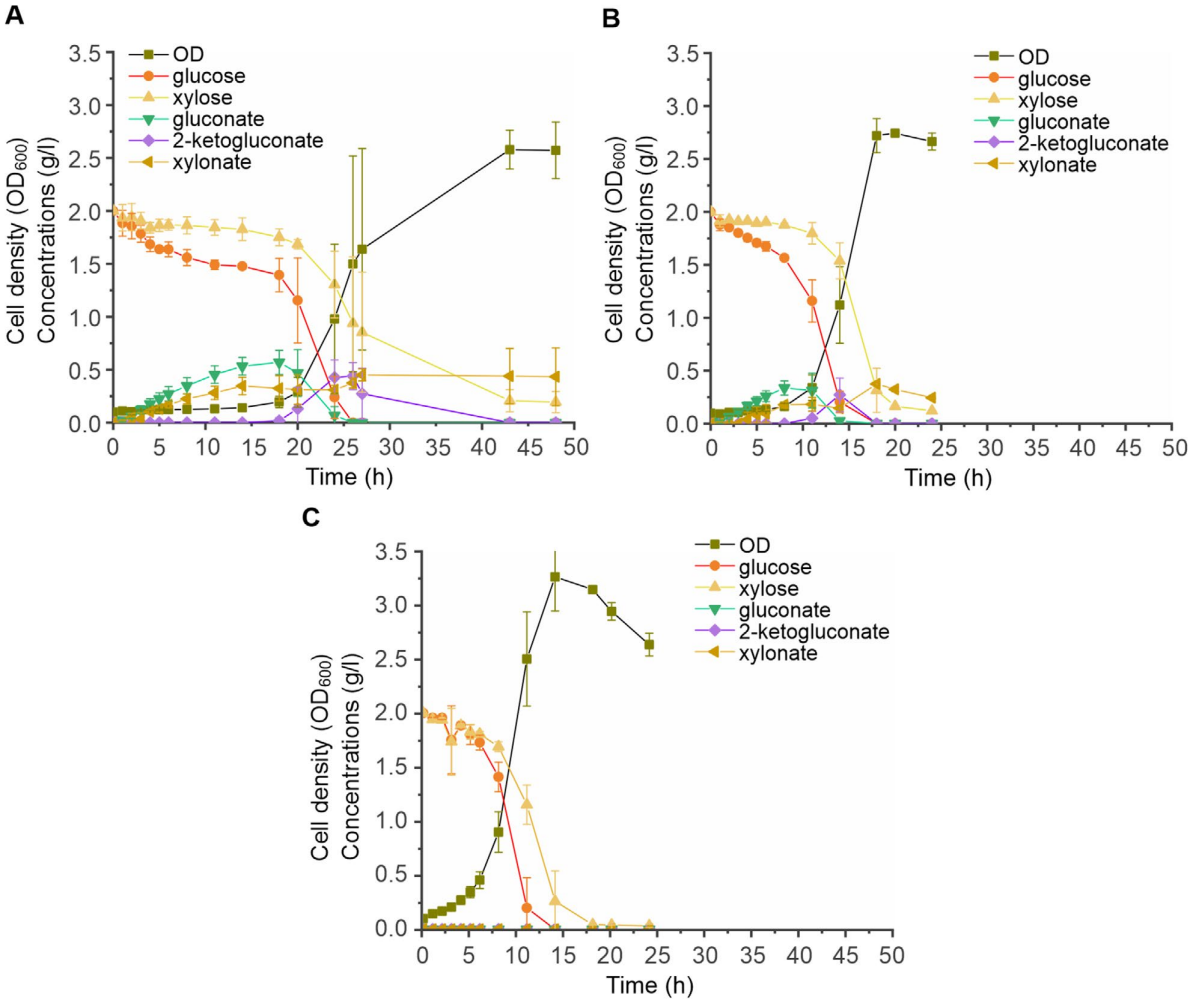
Co‐utilization of glucose and xylose by engineered *Pseudomonas putida* strains in shaken flasks. Cultivation was carried out in minimal M9 medium supplemented with glucose and xylose (2 g/L each), Km, Sm and 3‐methylbenzoate (25 μM), in a total volume of 50 mL in a 500‐mL Erlenmeyer flask. (A) EM42 XYL, (B) EM42 XYL *oprB*‐I, (C) EM42 ΔΔ XYL. Shown are means ± standard deviations from three (*n* = 3) biological replicates.

Glucose and xylose are mostly found together in lignocellulosic hydrolysates, which makes their simultaneous utilization by a production organism a must for viable lignocellulose biotechnologies (Dvořák et al. 2024; Narisetty et al. 2021; Ling et al. 2022). We compared EM42 XYL, EM42 XYL *oprB*‐I, and EM42 ΔΔ XYL (strain PD855 in Dvořák et al. 2024) in flask experiments, and we analysed concentrations of sugars and their oxidative metabolism intermediates (Figure 5). These strains were selected because EM42 XYL *oprB*‐I grows well on each of the two substrates (Figure 4), and EM42 ΔΔ XYL is the best xylose‐growing strain we constructed thus far (Dvořák et al. 2024).

The EM42 XYL strain (Figure 5A) utilized glucose faster than xylose, but the two substrates were consumed simultaneously—without diauxic shift. Glucose was oxidized to gluconate and 2‐ketogluconate, which were eventually used by the cells. The order of the respective concentration peaking corresponds to previous observations by other authors (Volke et al. 2023). Xylose was partially oxidized to the dead‐end product xylonate, as previously reported (Dvořák and de Lorenzo 2018; Dvořák, Bayer, and de Lorenzo 2020), causing a lower maximum OD_600_ of the EM42 XYL cell culture compared to the cultures of strain EM42 ΔΔ XYL, which completely utilized both glucose and xylose in 18 h (Figure 5C). In EM42 XYL *oprB*‐I cultures (Figure 5B), the products of oxidative glucose and xylose metabolism were still detected, but their concentrations were lower compared to EM42 XYL. Overall sugar utilization and growth was faster in EM42 XYL *oprB*‐I compared to EM42 XYL (the EM42 XYL *oprB*‐I cultures reached maximum OD_600_ already at 18 h compared to > 27 h in the case of EM42 XYL cultures). The highest maximal OD_600_ in the shortest time (14 h) and the fastest glucose/xylose co‐utilization was accomplished by EM42 ΔΔ XYL, the reverse‐engineered *P. putida* strain designed for the efficient growth on xylose in our former study (Dvořák et al. 2024, PD855).

## 4 Discussion

Substrate uptake is as crucial for efficient catabolism as the parameters of downstream enzymes. In agreement with previous reports, we show that OprB porins are important mediators for glucose uptake, but not the only ones (Saravolac et al. 1991; van den Berg 2012; Shrivastava et al. 2011; Wylie and Worobec 1995; Chevalier et al. 2017; Adewoye et al. 1998). We identify this in *P. putida* KT2440, and specifically in its two genome‐streamlined derivatives, EM42 and EM42 Δ*gcd*Δ*hexR*. The knockout of three other putative porin genes (CPGs: PP_0504, PP_2662 and PP_2702)—selected together with *oprB*‐I, *oprB*‐II and *oprB*‐III out of the initial set of 38 annotated candidate porin genes—did not affect growth on glucose under the tested conditions (Figure 2); however, we cannot exclude their importance for other substrates. The relevance of this topic warrants searches for other possible outer‐membrane sugar uptake routes in *P. putida* in future studies. It is noteworthy, in this context, that our initial bioinformatic analysis revealed that several genes annotated as porins likely do not form a pore. This is presumably not a result of incorrect annotation, as our investigations in other bacteria showed that almost all proteins annotated as porins do indeed form a pore (Table S4). In *P. putida* , most of the genes in question (PP_0773, PP_1122, PP_1128 and PP_4669) belong to the OmpA protein family based on sequence similarity; however, our analyses revealed that they are lipoproteins. Lipoproteins have numerous functions in bacteria, including cell wall metabolism, cell division, transmembrane signal transduction, antibiotic resistance and adhesion to host tissues during infection (Nakayama et al. 2012). Sugar transport facilitated by lipoproteins was, to the best of our knowledge, only reported in Gram‐positive bacteria (Sutcliffe et al. 1993; Sutcliffe and Hutchings 2007). Part of their sequence is shared with porins, suggesting that in *P. putida*, the evolutionary pressure that led to porin diversification helped create a set of proteins with functions that extend beyond the transport of molecules. The majority of the candidate genes (23 out of 38) were identified as members of the OprD family, which comprises amino‐acid‐specific porins. This is unsurprising, given that amino acids constitute an important component of plant root exudates—the natural carbon source for *P. putida* (Molina et al. 2020).

**FIGURE 6:**
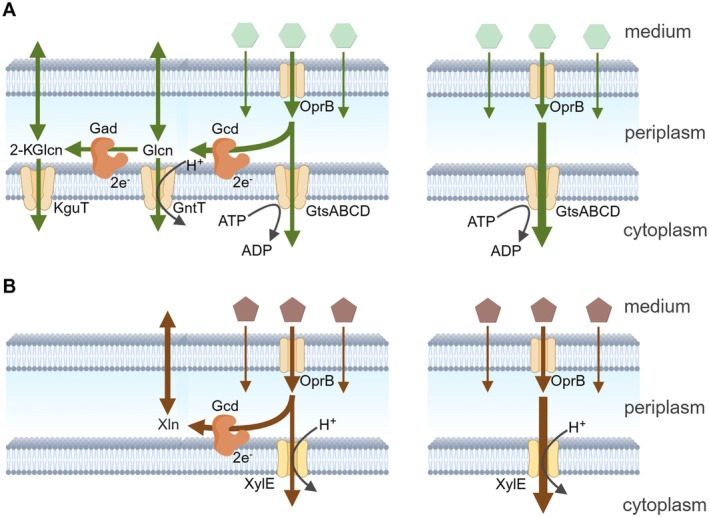
Glucose (A) and xylose (B) uptake routes in *Pseudomonas putida* EM42 (left) and EM42 Δ*gcd*Δ*hexR* (right) strains. (A) In *P. putida* EM42 (left), after crossing the outer phospholipid bilayer through OprB porins and other non‐specific channels (indicated by narrow green arrows), glucose (green hexagons) is either directly taken up via the ATP‐dependent ABC transporter GtsABCD or oxidized in the periplasm to gluconate (Glcn) and then to 2‐ketogluconate (2‐KGlcn) via glucose dehydrogenase (Gcd) and gluconate dehydrogenase (Gad). Glcn and 2‐KGlcn are transported into the cytoplasm via GntT (gluconate permease, glucose:H^+^ symporter) and KguT (unconfirmed transport mechanism), respectively, or released into the medium through unknown outer‐membrane channels. The electrons freed by the membrane‐bound dehydrogenases Gcd and Gad are transferred to the respiratory chain, contributing to the energetic budget of the cell. The direct and oxidative routes function in coordination; transport through the high‐affinity ABC transporter dominates at low glucose concentrations, while the oxidative path prevails as glucose concentrations increase. In *P. putida* EM42 Δ*gcd*Δ*hexR* (right), the oxidative route is absent, and the entire glucose flux is directed to GtsABCD. (B) In *P. putida* EM42 (left), after crossing the outer phospholipid bilayer through OprB porins and other non‐specific channels (indicated by narrow brown arrows), xylose is either directly taken up via the xylose:H^+^ symporter XylE or oxidized in the periplasm to xylonate (Xln) by the promiscuous glucose dehydrogenase Gcd. Both routes presumably share the xylose flux across different sugar concentrations; the dominance of either depends on the periplasmic xylose concentration and the relative abundance of Gcd and XylE. Xln is not utilized by the cells and is released into the medium through unknown outer‐membrane channel(s). In *P. putida* EM42 Δ*gcd*Δ*hexR* (right), the oxidative route is absent and the entire xylose flux is directed to XylE. Note: The Gcd reaction produces gluconolactone or xylonolactone, which is then hydrolyzed to gluconate or xylonate, respectively, by native lactonase or via spontaneous hydrolysis; this step is not shown. Components in the scheme are not to scale. Created with BioRender.com.

The status of the rhizosphere as the natural habitat of *P. putida* , alongside the organism's preference for organic and amino acids over carbohydrates, may also explain the relatively low glucose concentration (1 g/L) required to support the maximal growth rate observed in this study. Glucose concentration in the rhizosphere is expected to be low—below 1 mM (0.18 g/L) in most cases (Kuzyakov and Jones 2006). Studying the growth of *P. putida* and its *oprB* porin mutants at various glucose concentrations starting as low as 0.1 g/L helped us create a more complex picture. After glucose enters the periplasm, it is either transported to the cytoplasm by GtsABCD and phosphorylated by Glk or oxidized by Gcd (Figures 1 and 6A). Gts is a high‐affinity ATP‐dependent transporter (Wylie and Worobec 1995; Midgley and Dawes 1973), and because glucose concentration is low under natural conditions, direct glucose transport and phosphorylation is the primary metabolic route for glucose uptake in *P. putida* (Latrach Tlemçani et al. 2008; Whiting et al. 1976a, 1976b; Lessie and Phibbs 1984). This claim seemingly contradicts findings by Nikel et al. (2015) and Volke et al. (2023), who show that upon entering the cell, glucose is predominantly oxidized at a pace inversely correlated with growth rate. However, this stands true in laboratory conditions where glucose is not a limiting nutrient (at least not from the beginning of the cultivation). Both pathways, however, operate simultaneously, and the balance depends on several factors, such as the regulatory state and growth rate, as well as glucose concentration (Volke et al. 2023). With increasing Gts saturation in the wild‐type cells, the oxidation pathway, initiated with the low‐affinity enzyme Gcd (apparent *K*_m_ to glucose determined by An and Moe (2016) is 4.9 mM, i.e., ~0.9 g/L), starts to dominate (Daddaoua et al. 2014). Production of gluconate is advantageous on several levels: it can generate reducing power in the form of NAD(P)H, protecting against oxidative stress (Molina et al. 2020; Volke et al. 2023), it helps retrieve more nutrients (iron, magnesium, phosphate) from the environment (Buch et al. 2008; Uroz et al. 2020; Sasnow et al. 2016), and it boosts expression of GtsABCD via the GtrS/GltR regulator, promoting the phosphorylation pathway again (Daddaoua et al. 2014). Therefore, both routes must work in coordination.

Our data indicate that glucose transport through OprB porins is more prevalent at low, close to natural, sugar concentrations when Gts and phosphorylation route dominate. At the lowest glucose concentration used (0.1 g/L), the maximal specific growth rates of the EM42 and EM42 ΔΔ strains were reduced by 67% and 69%, respectively, after *oprB* gene knockouts (Figure S1A and Table S6). At 1.0 g/L, the reductions were only 36% and 44%, respectively (Figure 2 and Table S5). At high sugar concentrations (above 0.1 g/L) typical of laboratory or industrial settings, the transport of glucose can be dominated by facilitated diffusion through other, likely non‐sugar‐specific, outer membrane porins. The effect of the knockout of the *oprB* genes was more pronounced in the double‐deletion strain EM42 ΔΔ than in EM42 in all tested glucose concentrations. At higher glucose concentration of 10 g/L, EM42 ΔΔ might suffer from osmotic stress, and OprB inactivation can magnify the negative effect. The *gcd* deletion may lower the enzymatic ‘pull’ of glucose into the periplasm, which can exacerbate the slower substrate uptake in the Δ*oprB* strain. This could further delay the equilibration of sugar concentrations between the intracellular and extracellular environments. In addition, Gcd contributes to electron management and ATP generation in *P. putida* cells (Molina et al. 2020) (Figure 6A,B). The Δ*gcd* strain, therefore, may have less ATP, while all glucose must be processed via an ATP‐dependent Gts transporter and phosphorylation route. Possible sugar‐phosphate stress from accumulated glucose‐6‐phosphate might exacerbate these effects (Boulanger et al. 2021; Elmore et al. 2020).

The increased stress experienced by the EM42 ΔΔ strains at higher glucose concentrations is also reflected in their growth kinetics; these were best described by a substrate inhibition model, in contrast to the EM42‐derived strains, which followed traditional Monod kinetics (Figure S2). In these experiments, *P. putida* also showed two‐fold higher half‐saturation constant (*K*_S_) values for glucose compared to *E. coli* (Table S7). We attribute this difference to variations in outer membrane permeability, which is a key distinguishing factor between the bacterial species (Yoshimura and Nikaido 1982). Our measurements show an increase in *K*_S_ values in strains with knocked‐out OprB porins compared to controls, in agreement with lower membrane permeability (Figure S2 and Table S7).

Accordingly, the positive effect of complementing *oprB* knockouts with overexpressed *oprB*‐I was evident in *P. putida* EM42 ΔΔ, but not in EM42 (Figure 3 and Table S5). The increased glucose flux through OprB‐I may partially compensate for the loss of Gcd‐mediated metabolic pull in the former strain. In EM42 Δ*oprB* XYL, the metabolic burden imposed by the additional pSEVA2213 plasmid carrying three exogenous *xyl* genes could outweigh the advantage conferred by *oprB*‐I overexpression (Demko et al. 2019; Dvořák et al. 2024; Dvořák and de Lorenzo 2018). In contrast, overexpressing *oprB*‐I in strains with active OprB porins proved more beneficial in EM42 than in EM42 ΔΔ. The addition of OprB‐I molecules to the outer membrane of strain EM42 XYL *oprB*‐I increased its growth rate by ~25%–30% through accelerated glucose uptake and reduced gluconate and 2‐ketogluconate accumulation compared to the parental strain EM42 XYL (Figure 4B, Table S5, Figure S4B,C). We can try to infer more detailed information on the functioning of OprB porins from their genome context. The *oprB*‐I forms an operon with *gtsABCD* genes, forming the direct phosphorylation pathway. In conditions where this is the only available pathway for glucose utilization (EM42 ΔΔ strains), OprB‐I can supplement the function of all three knocked‐out OprB porins, as can be seen from the complementation experiment (Figure 3B). This experiment and *oprB*‐I overexpression in EM42 XYL *oprB*‐I suggest that OprB‐I is the major glucose uptake porin in *P. putida* . The *oprB*‐II gene forms an operon with the glucose dehydrogenase gene *gcd* and may represent a promising target for future studies focused on the outer membrane transport of the oxidative pathway metabolites, gluconate and 2‐ketogluconate. The last *oprB* gene, *oprB*‐III, does not form an operon with another gene and the OprB‐III porin shows only about 32% similarity (on amino acid level) with OprB‐I and OprB‐II (Sequence S2 in Supporting Information) so its specificity might have evolved towards a different substrate. A targeted analysis will be necessary to decipher the specific function of OprB‐III.

Further, our results suggest that the OprB porins in *P. putida* are not very substrate specific. Their manipulation affects, besides glucose, the transport of gluconate, citrate and most importantly, xylose, a non‐native substrate for *P. putida* (Figure 2C, Table S5, Figures S3 and S4). Porins of pseudomonads were described as more specific than the so‐called general porins of *E. coli* (Molina et al. 2020); however, it has previously been shown that they enable the passage of a spectrum of carbohydrates, including sucrose, maltose, arabinose and fructose (Saravolac et al. 1991; Shrivastava et al. 2011). We propose a similarly relaxed specificity for *P. putida* . In *P. putida* EM42 XYL, xylose is oxidized to a dead‐end product xylonate by the promiscuous Gcd in the periplasm (Figures 1 and 6B) (Dvořák and de Lorenzo 2018; Dvořák et al. 2024), resulting in very poor growth. Intriguingly, inactivation of OprB porins (EM42 ΔoprB XYL) proved advantageous in this strain background (Figure 2C and Table S5). We argue that the effect of knockouts was twofold. First, we hypothesize that this may have resulted in a lower periplasmic concentration of xylose; this would potentially favour transport through the XylE sugar‐H^+^ symporter system and entry into the heterologous isomerase pathway over oxidation by Gcd. Gcd has even lower affinity to non‐native substrate xylose than to glucose (Dvořák et al. 2024; Dvořák and de Lorenzo 2018; An and Moe 2016), while the apparent *K*_m_ of XylE to xylose is below 0.5 mM (< 0.08 g/L) (Sumiya et al. 1995). Second, knockouts of the oprB genes might have freed some capacity of protein secretion machinery for other molecules, particularly for XylE. The XylE transporter and the balancing of its amount have been repeatedly shown to be critical factors for efficient semi‐synthetic xylose metabolism in *P. putida* (Elmore et al. 2020; Ling et al. 2022; Dvořák et al. 2024). The latter hypothesis is further supported by experiment with the EM42 ΔCPG XYL strain, in which the candidate CPG porins were inactivated and the strain exhibited improved growth on xylose comparable to that observed for EM42 ΔoprB XYL (Figure 2C), and by the analysis of previously obtained proteomic dataset (Supporting Information 2 in Dvořák et al. 2024 and PRIDE dataset identifier PXD047537), which indicates relatively high abundance of OprB‐I, OprG and PP‐2662 porins in the outer membrane of xylose‐grown *P. putida* .

The results from the shake‐flask experiments also support the above hypotheses: slower xylose oxidation to xylonate during the initial culture phase, together with more efficient xylose utilization compared with EM42 XYL, indicates greater involvement of the XylE transporter in EM42 Δ*oprB* XYL (Figure S4E).

Although both *oprB* inactivation and *oprB*‐I overexpression significantly improved growth on xylose in the EM42 XYL strain (Figures 2C and 4C), and EM42 XYL *oprB*‐I showed slower xylonate formation and more efficient xylose utilization comparable to EM42 XYL (Figure S4F), the underlying mechanisms are likely distinct. The mechanism is particularly difficult to explain for EM42 XYL *oprB*‐I. At this point, we can only propose that enhanced xylose influx into the periplasm through overproduced OprB‐I was coupled with more balanced *xylABE* expression, possibly due to a ribosome‐sequestering effect (Qian et al. 2017) caused by *oprB*‐I positioned downstream of *xylABE*. Together, these effects may have favoured greater use of the direct phosphorylation route over Gcd‐mediated oxidation for xylose utilization.

In contrast to EM42 XYL, EM42 ΔΔ XYL with functional porins grows well on xylose, because Gcd is absent, and all xylose enters the xylose isomerase route and pentose phosphate pathway (Figure 6B). However, following *oprB* inactivation, the EM42 ΔΔ Δ*oprB* XYL strain completely lost its ability to grow, unlike EM42 ΔΔ XYL and EM42 ΔΔ ΔCPG XYL (Figure 2C and Table S5). Here, the similar factors proposed above for EM42 ΔΔ growing on glucose could come into play: the reduced enzymatic ‘pull’ of xylose into the periplasm and decreased ATP generation due to the absence of Gcd could exacerbate the limited flux of sugar across the outer membrane and the metabolic burden from overexpressed exogenous enzymes and XylE transporter (Demko et al. 2019), respectively. Both negative effects in EM42 ΔΔ Δ*oprB* XYL were neutralized by complementation with *oprB*‐I (Figure 3C and Table S5). As observed with glucose, the expression of *oprB*‐I alone in EM42 ΔΔ Δ*oprB* XYL *oprB*‐I was sufficient to restore growth. This restoration likely resulted from enhanced xylose flux and, potentially, reduced expression of the *xylABE* genes positioned upstream of the cloned *oprB*‐I through a ribosome sequestering effect (Qian et al. 2017), thereby lowering the metabolic burden. Overexpression of *oprB*‐I in the EM42 ΔΔ XYL background, which already contains functional OprB porins, did not provide any additional benefit (Figure 4C).

In line with the prior cultivation experiments using individual sugars, co‐utilization of glucose and xylose by engineered *P. putida* strains confirmed the benefits of *oprB*‐I overexpression. Regarding the growth kinetics, EM42 ΔΔ XYL, currently the most efficient rationally prepared xylose‐utilizing strain in our laboratory (Dvořák et al. 2024), outperformed the two other strains (Figure 5). Even so, EM42 XYL *oprB*‐I yielded a remarkable result. We demonstrate that the dynamics of simultaneous glucose and xylose uptake can be substantially improved even in *P. putida* cells with intact glucose dehydrogenase by altering OprB porin levels. Concentration profiles suggest that the oxidative pathway is utilized less in EM42 XYL *oprB*‐I, potentially favouring direct phosphorylation routes for glucose and xylose. Competition of glucose and xylose for a single Gcd active site can contribute to this effect (Dvořák, Kováč, and de Lorenzo 2020).

Taken together, we show that three OprB porins play an important role in substrate utilization in *P. putida* . They are more relevant in the double‐deletion strain EM42 Δ*gcd* Δ*hexR*, in which facilitated sugar influx through porins can compensate for the absence of periplasmic metabolic pull exerted by Gcd. Overexpression of *oprB*‐I improves the growth of *P. putida* EM42 on glucose and, intriguingly, on the non‐native substrate xylose, as well as on glucose‐xylose mixtures. Inactivation of OprB porins also affects the utilization of other substrates, such as citrate or gluconate. More complex studies encompassing detailed metabolomic, proteomic and fluxomic analyses will be necessary to better understand the dynamic effects of porin manipulations in *P. putida* and other Gram‐negative bacteria. A comprehensive evaluation of OprB porin knockouts and overexpression in mock and real lignocellulosic hydrolysates containing industrially relevant sugar concentrations, diverse glucose‐to‐xylose ratios and typical inhibitory compounds (e.g., furan derivatives, acetate and phenolics) will be necessary to assess the industrial relevance of the current findings. Nevertheless, we believe that already the data presented here and elsewhere warrant that porins will not be further overlooked in metabolic engineering and biotechnology studies, as their manipulation can lead to superior‐performing bacterial strains (Ladkau et al. 2016; Löwe et al. 2020; D'Arrigo et al. 2019).

## Author Contributions

**Pavel Dvořák:** conceptualization, investigation, funding acquisition, writing – original draft, writing – review and editing, supervision, visualization. **Tibor Botka:** supervision, methodology, investigation, writing – review and editing. **Barbora Popelářová:** investigation, methodology, conceptualization, writing – original draft, writing – review and editing, visualization, formal analysis, data curation. **Pablo I. Nikel:** supervision, funding acquisition, writing – review and editing. **Nicolas T. Wirth:** supervision, methodology, writing – review and editing. **Daniel C. Volke:** supervision, writing – review and editing.

## Funding

This project was funded by the Czech Science Foundation (project registration number 25‐16845S). D.C.V. and P.I.N. gratefully acknowledge financial support by the Novo Nordisk Foundation through grant NNF24SA0100980. CIISB, Instruct‐CZ Centre of Instruct‐ERIC EU consortium, funded by MEYS CR infrastructure project LM2023042 and European Regional Development Fund‐Project ‘Innovation of Czech Infrastructure for Integrative Structural Biology’ (No. CZ.02.01.01/00/23_015/0008175), is gratefully acknowledged for the financial support of the measurements at the CEITEC Proteomics Core Facility. Computational resources were provided by the e‐INFRA CZ project (ID: 90254), supported by MEYS CR.

## Conflicts of Interest

The authors declare no conflicts of interest.

## Supporting information


**Table S1:** Spacers used for the functional knockout of selected porin genes.
**Table S2:** Positions of premature STOP codons in porin genes introduced by the base editor technology.
**Table S3:** Primers used in this study.
**Table S4:** Porins of selected Gram‐negative bacteria. The ability of the protein to form a pore (Y = Yes, *N* = No) was determined based on published or predicted tertiary structures.
**Table S5:** Growth rates of *Pseudomonas putida* strains on various substrates (used substrate concentration 1 g/L) as determined in 48‐well plate format.
**Table S6:** Growth rates of selected *Pseudomonas putida* strains cultivated in M9 medium with 0.1 g/L of glucose. Growth rates were calculated manually as means ± standard deviations from four (*n* = 4) biological replicates.
**Table S7:** Growth kinetic parameters determined using Monod equations. Bacterial strains were cultivated in M9 medium with glucose (0.1–10 g/L). Parameters were calculated using Prism from four (*n* = 4) biological replicates.
**Figure S1:** Effect of glucose concentration on growth of *Pseudomonas putida* porin mutant strains. Cultivation in minimal M9 medium supplemented with 0.1 (A), 0.25 (B), and 10 g/L (C) of glucose in 48‐well microtiter plate. Shown are means ± standard deviations from four (*n* = 4) biological replicates.
**Figure S2:** Growth kinetics of *Escherichia coli* BL21(DE3) (A), *Pseudomonas putida* EM42 strains (B), and EM42 ΔΔ strains (C) cultivated on minimal M9 medium supplemented with various glucose concentrations in 48‐well MTPs. Plotted are growth rates against glucose concentration, data were fitted in GraphPad Prism 10.6.1 (Dotmatics) using Monod‐type equations (see Methods). All graphs show means ± standard deviations from four (*n* = 4) biological replicates.
**Figure S3:** The effect of knockouts of OprB porins in *Pseudomonas putida* strains grown on citrate (A) or gluconate (B) in minimal M9 medium supplemented with 1 g/L of substrate in 48‐well MTP. EM42 strain (left graphs), EM42 ΔΔ strain (right graphs). Shown are means ± standard deviations from at least three (*n* = 3) biological replicates.
**Figure S4:** Cultivations of engineered *Pseudomonas putida* strains in shaken flasks. M9 medium was supplemented with 2 g/L of glucose (A–C) or xylose (D‐F) and kanamycin, total volume 50 mL in a 500 mL Erlenmeyer flask. (A) EM42 pSEVA2213, (B) EM42 XYL (C) EM42 XYL *oprB*‐I, (D) EM42 XYL, (E) EM42 Δ*oprB* XYL and (F) EM42 XYL *oprB*‐I. Shown are means ± standard deviations from two (*n* = 2) biological replicates. The bottom table summarizes the growth parameters of the *Pseudomonas putida* strains during the described cultivations. *μ*_max_ represents the maximum specific growth rate determined over the entire cultivation period. *Y*_X/S_ is the biomass yield, *q*_S_ is the biomass‐specific substrate uptake rate and *q*_P_ is the biomass‐specific production rate of gluconate (GLN, for glucose cultures) or xylonate (XLN, for xylose cultures). For the glucose cultures (A, B, C), *Y*_X/S_, *q*_S_ and *q*_P_ were calculated based on the initial 4 h of cultivation. For the xylose cultures (D–F), these parameters were determined for the initial 12 h.
**Sequence S1:** Sequence of plasmid pSEVA2213_*xylABE*_*oprB*‐I L3. Key elements are highlighted in colour: *neoR*, neomycin/kanamycin resistance gene; *oriT*, origin of transfer; *trfA*, replication initiation protein*; *oriV*, origin of replication*; *rrnB* T1, terminator*; EM7, promoter; *xylA*, xylose isomerase; *xylB*, xylulose kinase; *xylE*, xylose‐proton symporter; *oprB*‐I, porin; lambda t0, terminator. Plasmid elements marked with an asterisk (*) are encoded in reverse orientation.
**Sequence S2:** Multiple alignment of OprB amino acid sequences from *P. putida* EM42 using CLUSTALW (fast pairwise alignment; word size 1, window size 5, gap penalty 3). Conserved residues identified by ESPript 3.2 (Gouet et al. 1999) are highlighted by red background, similar residues (global score 0.6) are in red and framed in blue. Protein similarity based on the amino acid level calculated by blastp: OprB‐I and OprB‐II 71.2% (cover 100%), OprB‐I and OprB‐III 32.2% (cover 97%), OprB‐II and OprB‐III 32.1% (cover 98%).

**Data S1:** Supporting Information.

## Data Availability

The data that support the findings of this study are available in Supporting Information of this article. Whole‐genome sequences and raw sequencing data are deposited under NCBI BioProject PRJNA1431338. Raw data for all graphs and tables displayed in the manuscript are deposited as Source Data file in the Zenodo repository (https://zenodo.org/) under doi identifier: 10.5281/zenodo.20346027.
