# Supplementary material for: Mind the Porins: Differential Effects of Porin Knockouts and Overexpression on Glucose and Xylose Uptake and Utilization in Pseudomonas putida
Source: Microb Biotechnol. 2026 Jul 3;19(7):e70406. doi: 10.1111/1751-7915.70406 (PMC13332142; doi:10.1111/1751-7915.70406)
Supplement: Supplementary file 1 — Table S1: Spacers used for the functional knockout of selected porin genes. Table S2: Positions of premature STOP codons in porin genes introduced by the base editor technology. Table S3: Primers used in this study. Table S4: Porins of selected Gram‐negative bacteria. The ability of the protein to form a pore (Y = Yes, N = No) was determined based on published or predicted tertiary structures. Table S5: Growth rates of Pseudomonas putida strains on various substrates (used substrate concentration 1 g/L) as determined in 48‐well plate format. Table S6: Growth rates of selected Pseudomonas putida strains cultivated in M9 medium with 0.1 g/L of glucose. Growth rates were calculated manually as means ± standard deviations from four (n = 4) biological replicates. Table S7: Growth kinetic parameters determined using Monod equations. Bacterial strains were cultivated in M9 medium with glucose (0.1–10 g/L). Parameters were calculated using Prism from four (n = 4) biological replicates. Figure S1: Effect of glucose concentration on growth of Pseudomonas putida porin mutant strains. Cultivation in minimal M9 medium supplemented with 0.1 (A), 0.25 (B), and 10 g/L (C) of glucose in 48‐well microtiter plate. Shown are means ± standard deviations from four (n = 4) biological replicates. Figure S2: Growth kinetics of Escherichia coli BL21(DE3) (A), Pseudomonas putida EM42 strains (B), and EM42 ΔΔ strains (C) cultivated on minimal M9 medium supplemented with various glucose concentrations in 48‐well MTPs. Plotted are growth rates against glucose concentration, data were fitted in GraphPad Prism 10.6.1 (Dotmatics) using Monod‐type equations (see Methods). All graphs show means ± standard deviations from four (n = 4) biological replicates. Figure S3: The effect of knockouts of OprB porins in Pseudomonas putida strains grown on citrate (A) or gluconate (B) in minimal M9 medium supplemented with 1 g/L of substrate in 48‐well MTP. EM42 strain (left graphs), EM42 ΔΔ strain (right graphs) [file MBT2-19-e70406-s002.docx]

Supporting Information

**Mind the porins: Differential effects of porin knockouts and overexpression on glucose and xylose uptake and utilization in *Pseudomonas putida***

Barbora Popelářová^1^, Nicolas T. Wirth^2^, Daniel C. Volke^2^, Tibor Botka^1^, Pablo I. Nikel^2^, Pavel Dvořák^1^

*^1^Department of Experimental Biology, Faculty of Science, Masaryk University, Kamenice 753/5, 62500, Brno, Czech Republic.*

*^2^The Novo Nordisk Foundation Biotechnology Research Institute for the Green Transition (BRiGHT), Technical University of Denmark, 2800, Kongens Lyngby, Denmark*

* Corresponding author:

Pavel Dvořák

Department of Experimental Biology,

Faculty of Science, Masaryk University, Kamenice 735/5, Brno 62500, Czech Republic

Phone: +420 549 493 396, E-mail: pdvorak@sci.muni.cz

**Supplementary tables**

**Table S1**. **Spacers used for the functional knock-out of selected porin genes.**

| **Spacer name** | **Sequence (5'→3')** | **Targeted gene** |
| --- | --- | --- |
| *PP_1019*_A | GGT CAT CCA TTT GGA TTC GC | *PP_1019* |
| *PP_1019*_B | GCT TGA TCC ACA TCT GGG TC |  |
| *PP_1445*_A | GAG CAT CCA AGG GGA GTC GC | *PP_1445* |
| *PP_1445*_B | CCC AGT CGC CGA GCA TCC AA |  |
| *PP_3570*_A | GGC CGC CCC AGT CAC CGG TC | *PP_3570* |
| *PP_3570*_B | CGC CAT CAA CTC GAA GAA GA |  |
| *PP_0504*_A | TTC CAG CCG TAC GCT GGC GT | *PP_0504* |
| *PP_0504*_B | GTC GCT GCA GTA CTA CCC AA |  |
| *PP_2662*_A | CGC CAG TCG CGG GTG AAG AT | *PP_2662* |
| *PP_2662*_B | GCG CCA GTT CTA TGG CAC GG |  |
| *PP_2702*_A | CAC AGC ACC ACT CGG GTT TC | *PP_2702* |
| *PP_2702*_B | GGT ATC CCA GGT GCC CTC CA |  |

**Table S2**. **Positions of premature STOP codons in porin genes introduced by the base editor technology.**

| **Locus tag** | **Gene name** | **Gene (bp) /**  **Protein length (AA)** | **Signal sequence (AA)** | **Secretion pathway** | **Protein length after base editing (AA)**** | **Portion of native protein (%)** |
| --- | --- | --- | --- | --- | --- | --- |
| *PP_1019* | *oprB*-I | 1344/447 | 26 | Sec/SPI* | 9 | 2.14 |
| *PP_1445* | *oprB*-II | 1335/444 | 23 | Sec/SPI* | 9 | 2.14 |
| *PP_3570* | *oprB*-III | 1269/422 | 22 | Sec/SPI* | 13 | 3.25 |
| *PP_0504* | *oprG* | 684/227 | 22 | Sec/SPI* | 95 | 46.34 |
| *PP_2662* | Hypothetical protein | 1188/395 | 31 | Sec/SPI* | 37 | 10.16 |
| *PP_2702* | Porin | 1131/376 | 23 | Sec/SPI* | 41 | 11.61 |

* SPI, signal peptidase I; ** protein length without signal sequence; bp, base pair; AA, amino acids

**Table S3**. **Primers used in this study.**

| **Primer name** | **Sequence (5'→3')** | | **Purpose** |
| --- | --- | --- | --- |
| *PP_1019*_A-pos1-gg-for | ATC GAG GTC TCC GTG GGG TCA TCC ATT TGG ATT CGC GTT TTA GAG CTA GAA ATA GC | | Assembly of pBEC_*oprB* |
| *PP_1019*_B-pos2-gg-for | ATC GAG GTC TCC AGG TGC TTG ATC CAC ATC TGG GTC GTT TTA GAG CTA GAA ATA GC | |  |
| *PP_1445*_A-pos3-gg-for | ATC GAG GTC TCC GCG GGA GCA TCC AAG GGG AGT CGC GTT TTA GAG CTA GAA ATA GC | |  |
| *PP_1445*_B-pos4-gg-for | ATC GAG GTC TCC TTG TCC CAG TCG CCG AGC ATC CAA GTT TTA GAG CTA GAA ATA GC | |  |
| *PP_3570*_A-pos5-gg-for | ATC GAG GTC TCC TGT TGG CCG CCC CAG TCA CCG GTC GTT TTA GAG CTA GAA ATA GC | |  |
| *PP_3570*_B-pos6-gg-for | ATC GAG GTC TCC TTT CCG CCA TCA ACT CGA AGA AGA GTT TTA GAG CTA GAA ATA GC | |  |
| *PP_0504*_A-pos1-gg-for | ATC GAG GTC TCC GTG GTT CCA GCC GTA CGC TGG CGT GTT TTA GAG CTA GAA ATA GC | | Assembly of pBEC_CPG |
| *PP_0504*_B-pos2-gg-for | ATC GAG GTC TCC AGG TGT CGC TGC AGT ACT ACC CAA GTT TTA GAG CTA GAA ATA GC | |  |
| *PP_2662*_A-pos3-gg-for | ATC GAG GTC TCC GCG GCG CCA GTC GCG GGT GAA GAT GTT TTA GAG CTA GAA ATA GC | |  |
| *PP_2662*_B-pos4-gg-for | ATC GAG GTC TCC TTG TGC GCC AGT TCT ATG GCA CGG GTT TTA GAG CTA GAA ATA GC | |  |
| *PP_2702*_A-pos5-gg-for | ATC GAG GTC TCC TGT TCA CAG CAC CAC TCG GGT TTC GTT TTA GAG CTA GAA ATA GC | |  |
| *PP_2702*_B-pos6-gg-for | ATC GAG GTC TCC TTT CGG TAT CCC AGG TGC CCT CCA GTT TTA GAG CTA GAA ATA GC | |  |
| gRNA-Position1-gg-rev | ATC GAG GTC TCC ACC TTT AGC TGC CTA TAC GGC AGT | | Universal reverse primers for pBEC assembly |
| gRNA-Position2-gg-rev | ATC GAG GTC TCC CCG CTT AGC TGC CTA TAC GGC AGT | |  |
| gRNA-Position3-gg-rev | ATC GAG GTC TCC ACA ATT AGC TGC CTA TAC GGC AGT | |  |
| gRNA-Position4-gg-rev | ATC GAG GTC TCC AAC ATT AGC TGC CTA TAC GGC AGT | |  |
| gRNA-Position5-gg-rev | ATC GAG GTC TCC GAA ATT AGC TGC CTA TAC GGC AGT | |  |
| gRNA-lastposition-gg-rev | ATC GAG GTC TCC AAA CTT TCT TAG CTG CCT ATA CGG | |  |
| **Primer name** | **Sequence (5'→3')** | **Purpose** | |
| pR1 | TTA GAC TCT CGT TTG GAT TGC | | Colony PCR checking of correct assembly of pBEC |
| pR3 | ACT GCC GGT TCT CCG AAT TGC AG | |  |
| *PP_0504*_seq_FW | CTT ACT ATG GCG CGT GCA TC | | Sequencing primer for base edited gene *PP_0504* |
| *PP_0504*_seq_RV | CGT TTA CCA GCA GGT TGT CG | |  |
| *PP_1019*_seq_FW | CTT CAA AGG CCG TTG ACT CG | | Sequencing primer for base edited gene *PP_1019* |
| *PP_1019*_seq_RV | AGG TTG GAA GGG TTC TGC TC | |  |
| *PP_1445*_seq_FW | ACT GTT GCC CGT TCA ACA AC | | Sequencing primer for base edited gene *PP_1445* |
| *PP_1445*_seq_RV | AAG CGG CCG AAT TTC ACA TC | |  |
| *PP_2662*_seq_FW | TGC CTG AGC CAT AAG TCT CG | | Sequencing primer for base edited gene *PP_2662* |
| *PP_2662*_seq_RV | TTT CCT GGT AGG CCT TTC CG | |  |
| *PP_2702*_seq_FW | CAG GCG ACC AAG GTT TTA CG | | Sequencing primer for base edited gene *PP_2702* |
| *PP_2702*_seq_RV | CAG GTC AAG GTA AAA GGC GC | |  |
| *PP_3570*_seq_FW | TGT TCA TCG CAG GTA CCC AG | | Sequencing primer for base edited gene *PP_3570* |
| *PP_3570*_seq_RV | GTT TTC CTG GAT TGG CAG GC | |  |
| PS2 | CG GCA ACC GAG CGT TC | | Checking *oprB*-I subcloning into pSEVA2213_*xylABE*_*oprB*-I plasmid |
| *xylE* seq | GCT GGT GGC CCA TTT CC | |  |

**Table S4**. **Porins of selected Gram-negative bacteria.** The ability of the protein to form a pore (Y = Yes, N = No) was determined based on published or predicted tertiary structures.

| **Organism** | **Gene** | **Name** | **Porin** |
| --- | --- | --- | --- |
| *Escherichia coli* K-12 substr. MG1655 | *bglH* | carbohydrate-specific outer membrane porin | Y |
|  | *nmpC 2* | DLP12 prophage; putative outer membrane porin NmpC | N |
|  | *nmpC 1* | putative outer membrane porin protein | Y |
|  | *fadL* | long-chain fatty acid outer membrane channel / bacteriophage T2 receptor | Y |
|  | *ompC* | outer membrane porin C | Y |
|  | *ompF* | outer membrane porin F | Y |
|  | *uidC* | outer membrane porin family protein UidC | Y |
|  | *ompG* | outer membrane porin G | Y |
|  | *ompN* | outer membrane porin N | Y |
|  | *phoE* | outer membrane porin PhoE | Y |
|  | *pgaA* | partially deacetylated poly-β-1,6-N-acetyl-D-glucosamine export outer membrane porin | Y |
|  | *ompL* | putative outer membrane porin L | Y |
|  | *yfeN* | putative outer membrane porin YfeN | Y |
|  | *yddL* | putative uncharacterized protein YddL | N |
|  | *eaeH* | putative porin domain-containing protein EaeH | N |
|  | *yfaZ* | putative porin YfaZ | Y |
|  | *aqpZ* | water channel AqpZ | Y |
| *Klebsiella pneumoniae pneumoniae* ATCC 43816 KPPR1 | *RS10375* | porin | Y |
|  | *ompA* | porin OmpA | Y |
|  | *RS24740* | porin OmpC | Y |
|  | *ompC* | porin OmpC | Y |
|  | *ompK35* | porin OmpK35 | Y |
|  | *ompK37* | porin ompK37 | Y |
|  | *aqpZ* | aquaporin Z | Y |
|  | *RS22475* | carbohydrate porin | Y |
|  | *RS03340* | carbohydrate porin | Y |
|  | *RS16915* | carbohydrate porin | Y |
|  | *RS02975* | carbohydrate porin | Y |
|  | *RS17240* | carbohydrate porin | Y |
|  | *RS25030* | carbohydrate porin | Y |
|  | *RS13675* | carbohydrate porin | Y |
|  | *RS10180* | carbohydrate porin | Y |
|  | *RS21575* | carbohydrate porin | Y |
|  | *chiP* | chitoporin | Y |
|  | *hofQ* | DNA uptake porin HofQ | Y |
|  | *fhuA* | ferrichrome porin FhuA | Y |
|  | *ompK26* | KdgM family porin OmpK26 | Y |
|  | *RS10605* | maltoporin | Y |
|  | *RS15395* | maltoporin | Y |
|  | *RS17505* | MlP/aquaporin family protein | Y |
|  | *RS20170* | MlP/aquaporin family protein | Y |
|  | *RS20860* | OprD family outer membrane porin | Y |
|  | *phoE* | phosphoporin PhoE | Y |
|  | *pgaA* | poly-beta-1,6 N-acetyl-D-glucosamine export porin PgaA | Y |

**Table S4** continuing

| **Organism** | **Gene** | **Name** | **Porin** |
| --- | --- | --- | --- |
| *Xanthomonas oryzae* KXO85 | *RS19475* | porin | Y |
|  | *RS10285* | carbohydrate porin | Y |
|  | *RS12150* | OprO/OprP family phosphate-selective porin | Y |
|  | *RS19540* | OprO/OprP family phosphate-selective porin | Y |
|  | *RS23185* | OprO/OprP family phosphate-selective porin | possible |
|  | *pgaA* | poly-beta-1,6 N-acetyl-D-glucosamine export porin PgaA | Y |
|  | *RS05565* | TorF family putative porin | Y |

**Table S5.** **Growth rates of *Pseudomonas putida* strains on various substrates (used substrate concentration 1 g/L) as determined in 48-well plate format.**

| Substrate | Strain | Growth rate (h^-1^) |
| --- | --- | --- |
| Glucose | EM42 | 0.96 ± 0.07 |
|  | EM42 Δ*oprB* | 0.61 ± 0.03 |
|  | EM42 ΔΔ | 0.61 ± 0.02 |
|  | EM42 ΔΔ Δ*oprB* | 0.34 ± 0.06 |
|  | EM42 ΔCPG | 0.82 ± 0.07 |
|  | EM42 ΔΔ ΔCPG | 0.61 ± 0.03 |
|  | EM42 XYL | 0.54 ± 0.03 |
|  | EM42 Δ*oprB* XYL | 0.54 ± 0.04 |
|  | EM42 XYL *oprB*-I | 0.70 ± 0.03 |
|  | EM42 Δ*oprB* XYL *oprB*-I | 0.53 ± 0.04 |
|  | EM42 ΔΔ XYL | 0.48 ± 0.01 |
|  | EM42 ΔΔ Δ*oprB* XYL | 0.18 ± 0.01 |
|  | EM42 ΔΔ XYL *oprB*-I | 0.43 ± 0.03 |
|  | EM42 ΔΔ Δ*oprB* XYL *oprB*-I | 0.32 ± 0.01 |
| Xylose* | EM42 XYL | 0.01 ± 0.00 |
|  | EM42 Δ*oprB* XYL | 0.19 ± 0.02 |
|  | EM42 ΔCPG XYL | 0.22 ± 0.02 |
|  | EM42 ΔΔ XYL | 0.18 ± 0.01 |
|  | EM42 ΔΔ Δ*oprB* XYL | NA |
|  | EM42 ΔΔ ΔCPG XYL | 0.24 ± 0.01 |
|  | EM42 XYL *oprB*-I | 0.18 ± 0.01 |
|  | EM42 Δ*oprB* XYL *oprB*-I | 0.17 ± 0.00 |
|  | EM42 ΔΔ XYL *oprB*-I | 0.17 ± 0.00 |
|  | EM42 ΔΔ Δ*oprB* XYL *oprB*-I | 0.11 ± 0.00 |
| Gluconate | EM42 | 0.75 ± 0.03 |
|  | EM42 Δ*oprB* | 0.51 ± 0.04 |
|  | EM42 ΔΔ | 0.69 ± 0.04 |
|  | EM42 ΔΔ Δ*oprB* | 0.44 ± 0.01 |
| Citrate | EM42 | 0.68 ± 0.09 |
|  | EM42 Δ*oprB* | 0.60 ± 0.04 |
|  | EM42 ΔΔ | 0.70 ± 0.04 |
|  | EM42 ΔΔ Δ*oprB* | 0.40 ± 0.01 |

*, all strains also contain the pSEVA438_*tal* plasmid; ΔΔ, Δ*gcd* Δ*hexR*; CPG, candidate porin genes; XYL, strains with pSEVA2213_*xylABE* L3 plasmid; NA, not applicable

**Table S6**. **Growth rates of selected *Pseudomonas putida* strains cultivated in M9 medium with 0.1 g/L of glucose.** Growth rates were calculated manually as means ± standard deviations from four (n=4) biological replicates.

| Strain | Growth rate (h^-1^) |
| --- | --- |
| EM42 | 0.52 ± 0.06 |
| EM42 Δ*oprB* | 0.17 ± 0.01 |
| EM42 ΔCPG | 0.46 ± 0.03 |
| EM42 ΔΔ | 0.39 ± 0.09 |
| EM42 ΔΔ Δ*oprB* | 0.12 ± 0.04 |
| EM42 ΔΔ ΔCPG | 0.54 ± 0.12 |

**Table S7**. **Growth kinetic parameters determined using Monod equations.** Bacterial strains were cultivated in M9 medium with glucose (0.1 - 10 g/L). Parameters were calculated using Prism from four (n=4) biological replicates.

|  | *E. coli* BL21 (DE3) | *P. putida* EM42 | *P. putida* EM42 Δ*oprB* | *P. putida*  EM42 ΔΔ | *P. putida* EM42  ΔΔ Δ*oprB* |
| --- | --- | --- | --- | --- | --- |
| Best-fit values | | | | | |
| µ_max_ (h^-1^) | 0.56 | 0.94 | 0.68 | 0.66 | 0.86 |
| *K*_S_ (g/L) | 0.04 | 0.08 | 0.13 | 0.06 | 0.49 |
| *K*_i_ (g/L) | - | - | - | 31.11 | 1.15 |
| 95% confidence interval | | | | | |
| µ_max_ (h^-1^) | 0.54 – 0.59 | 0.89 – 0.99 | 0.63 – 0.74 | 0.61 – 0.73 | 0.58 – 1.94 |
| *K*_S_ (g/L) | 0.02 – 0.05 | 0.06 – 0.11 | 0.09 – 0.19 | 0.03 – 0.09 | 0.25 – 1.42 |
| *K*_i_ (g/L) | - | - | - | 17.14 – 81.00 | 0.37 – 2.41 |

µ_max_, maximum specific growth rate; K_S_, half-saturation constant; *K*_i_, inhibition constant

**Supplementary figures**


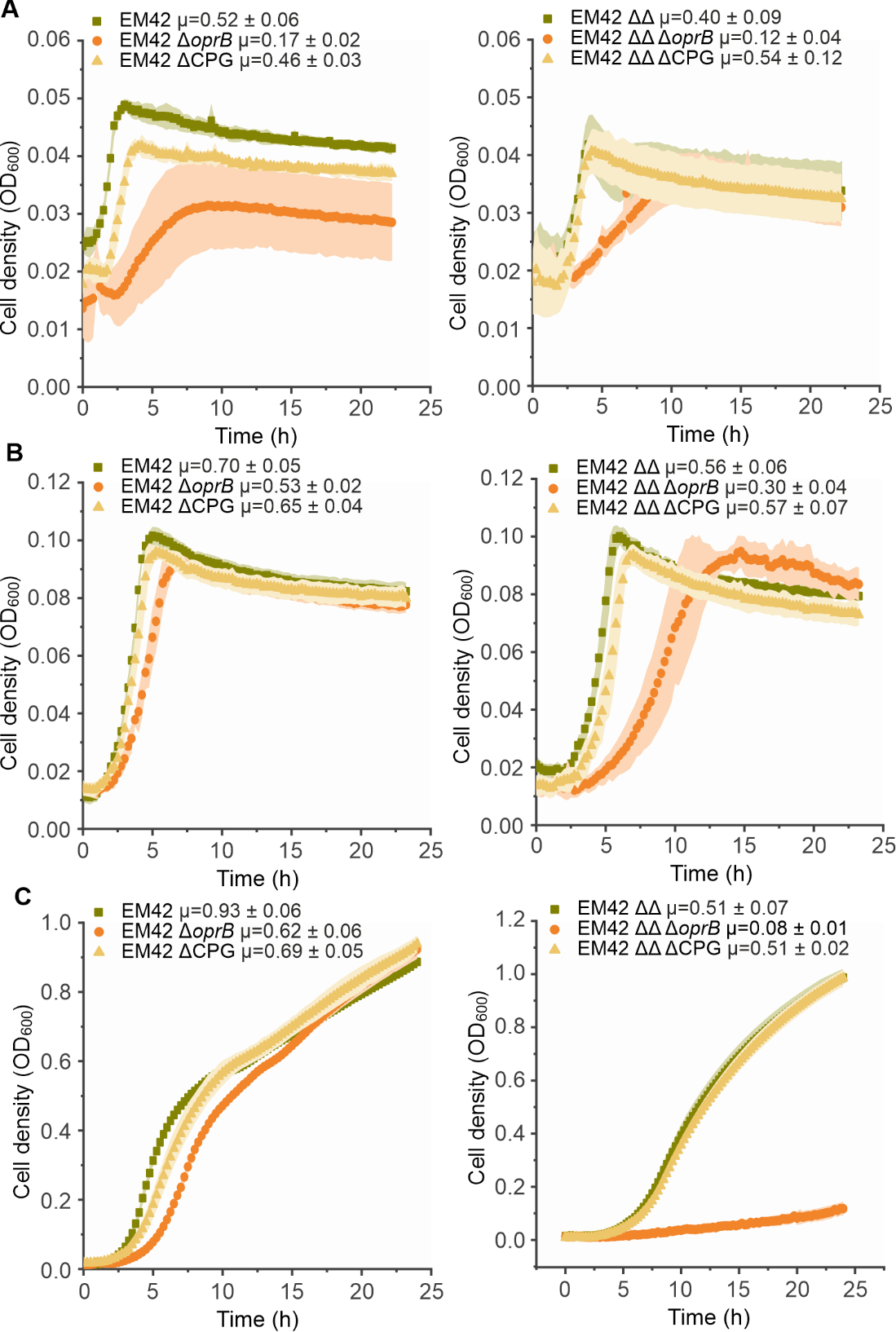


**Figure S1**. **Effect of glucose concentration on growth of *Pseudomonas putida* porin mutant strains.** Cultivation in minimal M9 medium supplemented with 0.1 (**A**), 0.25 (**B**), and 10 g/L (**C**) of glucose in 48-well microtiter plate. Shown are means ± standard deviations from four (n=4) biological replicates.

**
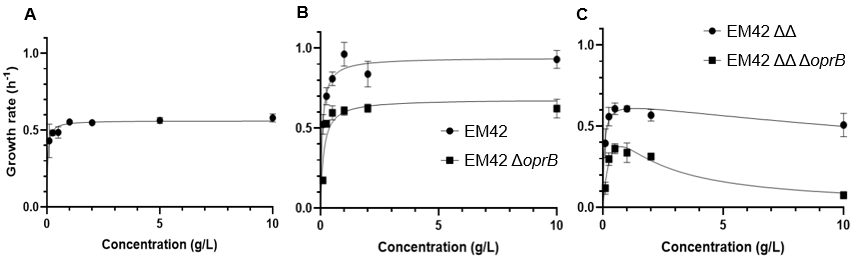
**

**Figure S2**. **Growth kinetics of *Escherichia coli* BL21(DE3) (A), *Pseudomonas putida* EM42 strains (B), and EM42 ΔΔ strains (C) cultivated on minimal M9 medium supplemented with various glucose concentrations in 48-well MTPs**. Plotted are growth rates against glucose concentration, data were fitted in GraphPad Prism 10.6.1 (Dotmatics) using Monod-type equations (see **Methods**). All graphs show means ± standard deviations from four (n=4) biological replicates.

**
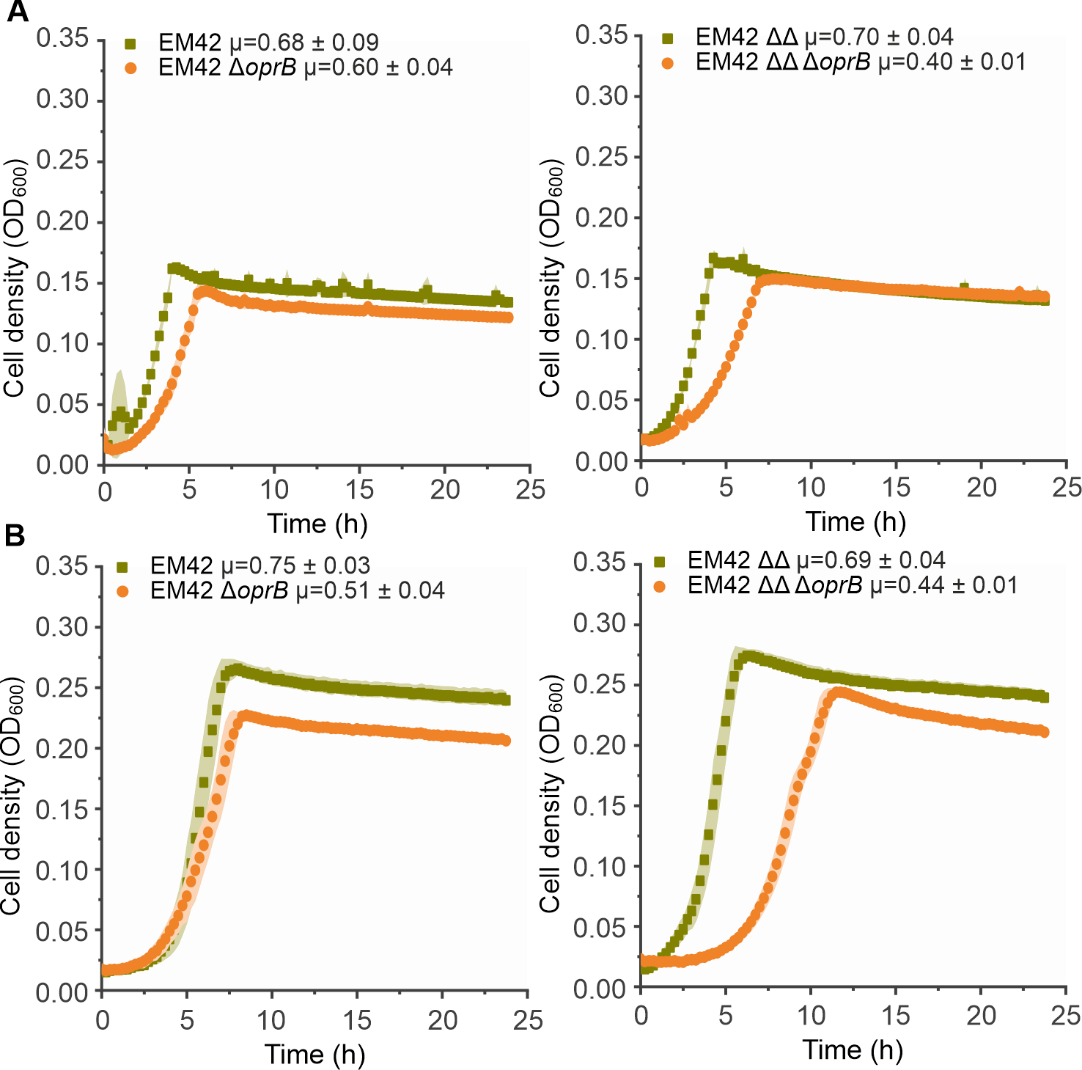
**

**Figure S3**. **The effect of knockouts of OprB porins in *Pseudomonas putida* strains grown on citrate (A) or gluconate (B) in minimal M9 medium supplemented with 1 g/L of substrate in 48-well MTP.** EM42 strain (left graphs), EM42 ΔΔ strain (right graphs). Shown are means ± standard deviations from at least three (n=3) biological replicates.

**
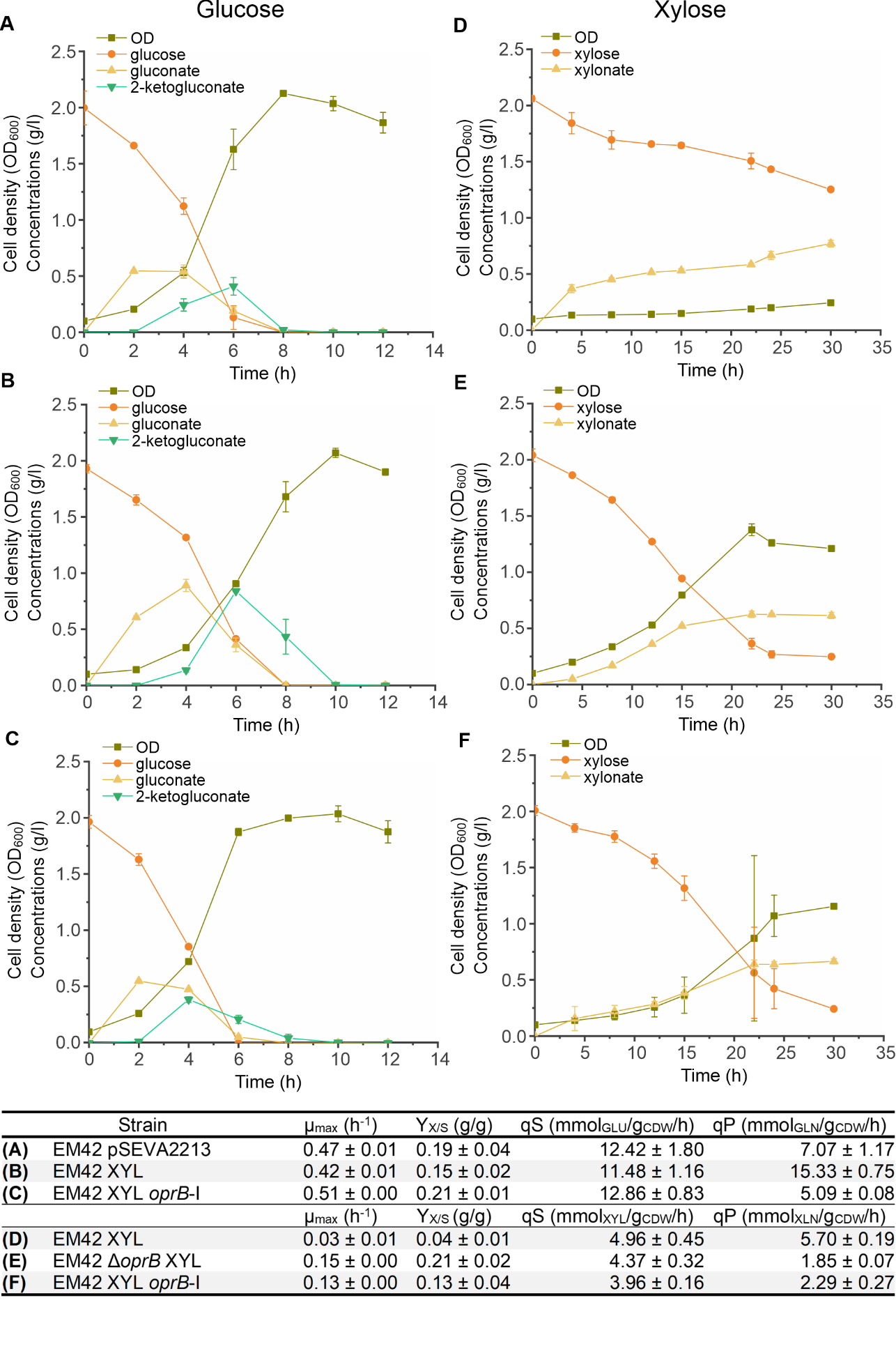
**

**Figure S4. Cultivations of engineered *Pseudomonas putida* strains in shaken flasks.** M9 medium was supplemented with 2 g/L of glucose (**A-C**) or xylose (**D-F**) and kanamycin, total volume 50 mL in a 500 mL Erlenmeyer flask. (**A**) EM42 pSEVA2213, (**B**) EM42 XYL (**C**) EM42 XYL *oprB*-I, (**D**) EM42 XYL, (**E**) EM42 Δ*oprB* XYL, (**F**) EM42 XYL *oprB*-I. Shown are means ± standard deviations from 2 (n=2) biological replicates. The bottom table summarizes the growth parameters of the *Pseudomonas putida* strains during the described cultivations. µ_max_ represents the maximum specific growth rate determined over the entire cultivation period. Y_X/S_ is the biomass yield, qS is the biomass-specific substrate uptake rate, and qP is the biomass-specific production rate of gluconate (GLN, for glucose cultures) or xylonate (XLN, for xylose cultures). For the glucose cultures (A, B, C), Y_X/S_x, qS and qP were calculated based on the initial four hours of cultivation. For the xylose cultures (D, E, F), these parameters were determined for the initial twelve hours.

**Supplementary sequences**

**Sequence S1**. Sequence of plasmid pSEVA2213_*xylABE*_*oprB*-I L3. Key elements are highlighted in color: *neoR*, neomycin/kanamycin resistance gene; *oriT*, origin of transfer; *trfA*, replication initiation protein*; *oriV*, origin of replication*; *rrnB* T1, terminator*; EM7, promoter; *xylA*, xylose isomerase; *xylB*, xylulose kinase; *xylE*, xylose-proton symporter; *oprB*-I, porin; lambda t0, terminator. Plasmid elements marked with an asterisk (*) are encoded in reverse orientation.

1 cctgggcgaa tttagcccga gcctgcaaaa acgtctgttt cagaaatatg gcattgataa

61 tccggatatg aacaaactgc aatttcatct gatgctggat gaatttttct aataattaat

121 tggaccgcgg tccgcgcgtt gtccttttcc gctgcataac cctgcttcgg ggtcattata

181 gcgatttttt cggtatatcc atcctttttc gcacgatata caggattttg ccaaagggtt

241 cgtgtagact ttccttggtg tatccaacgg cgtcagccgg gcaggatagg tgaagtaggc

301 ccacccgcga gcgggtgttc cttcttcact gtcccttatt cgcacctggc ggtgctcaac

361 gggaatcctg ctctgcgagg ctggccgtag gccggccgcg atgcaggtgg ctgctgaacc

421 cccagccgga actgacccca caaggcccta gcgtttgcaa tgcaccaggt catcattgac

481 ccaggcgtgt tccaccaggc cgctgcctcg caactcttcg caggcttcgc cgacctgctc

541 gcgccacttc ttcacgcggg tggaatccga tccgcacatg aggcggaagg tttccagctt

601 gagcgggtac ggctcccggt gcgagctgaa atagtcgaac atccgtcggg ccgtcggcga

661 cagcttgcgg tacttctccc atatgaattt cgtgtagtgg tcgccagcaa acagcacgac

721 gatttcctcg tcgatcagga cctggcaacg ggacgttttc ttgccacggt ccaggacgcg

781 gaagcggtgc agcagcgaca ccgattccag gtgcccaacg cggtcggacg tgaagcccat

841 cgccgtcgcc tgtaggcgcg acaggcattc ctcggccttc gtgtaatacc ggccattgat

901 cgaccagccc aggtcctggc aaagctcgta gaacgtgaag gtgatcggct cgccgatagg

961 ggtgcgcttc gcgtactcca acacctgctg ccacaccagt tcgtcatcgt cggcccgcag

1021 ctcgacgccg gtgtaggtga tcttcacgtc cttgttgacg tggaaaatga ccttgttttg

1081 cagcgcctcg cgcgggattt tcttgttgcg cgtggtgaac agggcagagc gggccgtgtc

1141 gtttggcatc gctcgcatcg tgtccggcca cggcgcaata tcgaacaagg aaagctgcat

1201 ttccttgatc tgctgcttcg tgtgtttcag caacgcggcc tgcttggctt cgctgacctg

1261 ttttgccagg tcctcgccgg cggtttttcg cttcttggtc gtcatagttc ctcgcgtgtc

1321 gatggtcatc gacttcgcca aacctgccgc ctcctgttcg agacgacgcg aacgctccac

1381 ggcggccgat ggcgcgggca gggcaggggg agccagttgc acgctgtcgc gctcgatctt

1441 ggccgtagct tgctggacta tcgagccgac ggactggaag gtttcgcggg gcgcacgcat

1501 gacggtgcgg cttgcgatgg tttcggcatc ctcggcggaa aaccccgcgt cgatcagttc

1561 ttgcctgtat gccttccggt caaacgtccg attcattcac cctccttgcg ggattgcccc

1621 ggaattaatt ccccggatcg atccgtcgat cttgatcccc tgcgccatca gatccttggc

1681 ggcaagaaag ccatccagtt tactttgcag ggcttcccaa ccttaccaga gggcgcccca

1741 gctggcaatt ccggttcgct tgctgtccat aaaaccgccc agtctagcta tcgccatgta

1801 agcccactgc aagctacctg ctttctcttt gcgcttgcgt tttcccttgt ccagatagcc

1861 cagtagctga cattcatccg gggtcagcac cgtttctgcg gactggcttt ctacgtggct

1921 gccatttttg gggtgaggcc gttcgcggcc gaggggcgca gcccctgggg ggatgggagg

1981 cccgcgttag cgggccggga gggttcgaga aggggggcac cccccttcgg cgtgcgcggt

2041 cacgcgcaca gggcgcagcc ctggttaaaa acaaggttta taaatattgg tttaaaagca

2101 ggttaaaaga caggttagcg gtggccgaaa aacgggcgga aacccttgca aatgctggat

2161 tttctgcctg tggacagccc ctcaaatgtc aataggtgcg cccctcatct gtcagcactc

2221 tgcccctcaa gtgtcaagga tcgcgcccct catctgtcag tagtcgcgcc cctcaagtgt

2281 caataccgca gggcacttat ccccaggctt gtccacatca tctgtgggaa actcgcgtaa

2341 aatcaggcgt tttcgccgat ttgcgaggct ggccagctcc acgtcgccgg ccgaaatcga

2401 gcctgcccct catctgtcaa cgccgcgccg ggtgagtcgg cccctcaagt gtcaacgtcc

2461 gcccctcatc tgtcagtgag ggccaagttt tccgcgaggt atccacaacg ccggcggccc

2521 tacatggctc tgctgtagtg agtgggttgc gctccggcag cggtcctgat cccccgcaga

2581 aaaaaaggat ctcaagaaga tcctttgatc ttttctacgg cgcgcccagc tgtctagggc

2641 ggcggatttg tcctactcag gagagcgttc accgacaaac aacagataaa acgaaaggcc

2701 cagtctttcg actgagcctt tcgttttatt tgatgccttt aattaattgt tgacaattaa

2761 tcatcggcat agtatatcgg catagtataa tacgacaagg tgaggaacta aacccctagg

2821 ccgcggccgc gcgaattcta gcaagaggaa tataccatgc aagcctattc tagcaagagg

2881 aatataccat gcaagcctat tttgaccagc tcgatcgcgt tcgttatgaa ggctcaaaat

2941 cctcaaaccc gttagcattc cgtcactaca atcccgacga actggtgttg ggtaagcgta

3001 tggaagagca cttgcgtttt gccgcctgct actggcacac cttctgctgg aacggggcgg

3061 atatgtttgg tgtgggggcg tttaatcgtc cgtggcagca gcctggtgag gcactggcgt

3121 tggcgaagcg taaagcagat gtcgcatttg agtttttcca caagttacat gtgccatttt

3181 attgcttcca cgatgtggat gtttcccctg agggcgcgtc gttaaaagag tacatcaata

3241 attttgcgca aatggttgat gtcctggcag gcaagcaaga agagagcggc gtgaagctgc

3301 tgtggggaac cgccaactgc tttacaaacc ctcgctacgg cgcgggtgcg gcgacgaacc

3361 cagatcctga agtcttcagc tgggcggcaa cgcaagttgt tacagcgatg gaagcaaccc

3421 ataaattggg cggtgaaaac tatgtcctgt ggggcggtcg tgaaggttac gaaacgctgt

3481 taaataccga cttgcgtcag gagcgtgaac aactgggccg ctttatgcag atggtggttg

3541 agcataaaca taaaatcggt ttccagggca cgttgcttat cgaaccgaaa ccgcaagaac

3601 cgaccaaaca tcaatatgat tacgatgccg cgacggtcta tggcttcctg aaacagtttg

3661 gtctggaaaa agagattaaa ctgaacattg aagctaacca cgcgacgctg gcaggtcact

3721 ctttccatca tgaaatagcc accgccattg cgcttggcct gttcggttct gtcgacgcca

3781 accgtggcga tgcgcaactg ggctgggaca ccgaccagtt cccgaacagt gtggaagaga

3841 atgcgctggt gatgtatgaa attctcaaag caggcggttt caccaccggt ggtctgaact

3901 tcgatgccaa agtacgtcgt caaagtactg ataaatatga tctgttttac ggtcatatcg

3961 gcgcgatgga tacgatggca ctggcgctga aaattgcagc gcgcatgatt gaagatggcg

4021 agctggataa acgcatcgcg cagcgttatt ccggctggaa tagcgaattg ggccagcaaa

4081 tcctgaaagg ccaaatgtca ctggcagatt tagccaaata tgctcaggaa cataatttgt

4141 ctccggtgca tcagagtggt cgccaggagc aactggaaaa tctggtaaat cattatctgt

4201 tcgacaaata acggctaact gtgcagtccg ttggcccggt tatcggtagc gataccgggc

4261 atttttttaa ggaacgatcg atatgtatat cgggatagat cttggcacct cgggcgtaaa

4321 agttattttg ctcaacgagc agggtgaggt ggttgcttcg caaacggaaa agctgaccgt

4381 ttcgcgcccg catccactct ggtcggaaca agacccggaa cagtggtggc aggcaactga

4441 tcgcgcaatg aaagctctgg gcgatcagca ttctctgcag gacgttaaag cattgggtat

4501 tgccggccag atgcatggag caaccttact ggatgctcaa caacgggtat tgcgccctgc

4561 cattttgtgg aacgacgggc gctgtgcgca agagtgcact ttgctggaag cgagagttcc

4621 gcaatcacga gtgattaccg gcaacctgat gatgcccgga tttactgcgc ctaaattgct

4681 atgggttcag cggcatgagc cggagatatt ccgtcaaatc gacaaagtat tattaccgaa

4741 agattacttg cgtctgcgta tgacggggga gtttgccagc gatatgtctg acgcagctgg

4801 caccatgtgg ctggatgtcg caaagcgtga ctggagtgac gtcatgctgc aggcttgcga

4861 cttatctcgt gaccagatgc ccgcattata cgaaggcagc gaaattactg gtgctttgtt

4921 acctgaagtt gcgaaagcgt ggggtatggc gacggtgcca gttgtcgcag gcggtggcga

4981 caatgcagct ggtgcagttg gtgtgggaat ggttgatgct aatcaggcaa tgttatcgct

5041 ggggacgtcg ggggtctatt ttgctgtcag cgaagggttc ttaagcaagc cagaaagcgc

5101 cgtacatagc ttttgccatg cgctaccgca acgttggcat ttaatgtctg tgatgctgag

5161 tgcagcgtcg tgtctggatt gggccgcgaa attaaccggc ctgagcaatg tcccagcttt

5221 aatcgctgca gctcaacagg ctgatgaaag tgccgagcca gtttggtttc tgccttatct

5281 ttccggcgag cgtacgccac acaataatcc ccaggcgaag ggggttttct ttggtttgac

5341 tcatcaacat ggccccaatg aactggcgcg agcagtgctg gaaggcgtgg gttatgcgct

5401 ggcagatggc atggatgtcg tgcatgcctg cggtattaaa ccgcaaagtg ttacgttgat

5461 tgggggcggg gcgcgtagtg agtactggcg tcagatgctg gcggatatca gcggtcagca

5521 gctcgattac cgtacgggag gggatgtggg gccagcactg ggcgcagcaa ggctggcgca

5581 gatcgcggcg aatccagaga aatcgctcat tgaattgttg ccgcaactac cgttagaaca

5641 gtcgcatcta ccagatgcgc agcgttatgc cgcttatcag ccacgacgag aaacgttccg

5701 tcgcctctat cagcaacttc tgccattaat ggcgtaagga tccctttaag aaggagatat

5761 acatatgaat acccagtata attccagtta tatattttcg attaccttag tcgctacatt

5821 aggtggttta ttatttggct acgacaccgc cgttatttcc ggtactgttg agtcactcaa

5881 taccgtcttt gttgctccac aaaacttaag tgaatccgct gccaactccc tgttagggtt

5941 ttgcgtggcc agcgctctga ttggttgcat catcggcggt gccctcggtg gttattgcag

6001 taaccgcttc ggtcgtcgtg attcacttaa gattgctgct gtcctgtttt ttatttctgg

6061 tgtaggttct gcctggccag aacttggttt tacctctata aacccggaca acacagtgcc

6121 tgtttatctg gcaggttatg tcccggaatt tgttatttat cgcattattg gcggtattgg

6181 cgttggttta gcctcaatgc tctcgccaat gtatattgcg gaactggctc cagctcatat

6241 tcgcgggaaa ctggtctctt ttaaccagtt tgcgattatt ttcgggcaac ttttagttta

6301 ctgcgtaaac tattttattg cccgttccgg tgatgccagc tggctgaata ctgacggctg

6361 gcgttatatg tttgcctcgg aatgtatccc tgcactgctg ttcttaatgc tgctgtatac

6421 cgtgccagaa agtcctcgct ggctgatgtc gcgcggcaag caagaacagg cggaaggtat

6481 cctgcgcaaa attatgggca acacgcttgc aactcaggca gtacaggaaa ttaaacactc

6541 cctggatcat ggccgcaaaa ccggtggtcg tctgctgatg tttggcgtgg gcgtgattgt

6601 aatcggcgta atgctctcca tcttccagca atttgtcggc atcaatgtgg tgctgtacta

6661 cgcgccggaa gtgttcaaaa cgctgggggc cagcacggat atcgcgctgt tgcagaccat

6721 tattgtcgga gttatcaacc tcaccttcac cgttctggca attatgacgg tggataaatt

6781 tggtcgtaag ccactgcaaa ttatcggcgc actcggaatg gcaatcggta tgtttagcct

6841 cggtaccgcg ttttacactc aggcaccggg tattgtggcg ctactgtcga tgctgttcta

6901 tgttgccgcc tttgccatgt cctggggtcc ggtatgctgg gtactgctgt cggaaatctt

6961 cccgaatgct attcgtggta aagcgctggc aatcgcggtg gcggcccagt ggctggcgaa

7021 ctacttcgtc tcctggacct tcccgatgat ggacaaaaac tcctggctgg tggcccattt

7081 ccacaacggt ttctcctact ggatttacgg ttgtatgggc gttctggcag cactgtttat

7141 gtggaaattt gtcccggaaa ccaaaggtaa aacccttgag gagctggaag cgctctggga

7201 accggaaacg aagaaaacac aacaaactgc tacgctgtaa aagcttcttt aagaaggaga

7261 tatacatatg gaacagcgca aacgcatcaa gacactggga tcgttggcct tgcttgcact

7321 tgtaggcagc agcggtacac aggctgccga ggctttttcc agcgaatcca aatggatgac

7381 cggcgactgg ggcggcaccc ggaccgagct gctggacaag ggctatgact tcaccctcga

7441 ttatgtgggt gaggtggctg gcaacctgca tggcggctac aacgacgaca agacggcacg

7501 ctacagcgac cagttcgccc tcggcgcgca tctggacttg cagaagatac tgggctggca

7561 tgatgccgag ttcaagctgg caatcaccga gcgaagcggt cgcaacctgt ccaacgaccg

7621 catcagcgac ccgcgcgccg ggcagttcag ctcggtgcag gaggtgtggg gccgtggcca

7681 gacctggcgc ctgacccaga tgtggatcaa gcagaagtac ttcgacggcg cgctggacgt

7741 gaaatttggc cgttttggcg agggcgagga cttcaacagc ttcccttgcg acttccagaa

7801 cctggccttc tgcggctcgc aggtgggcaa ctgggtgggc ggcatctggt acaactggcc

7861 ggtcagccag tgggcgctgc gggtgaagta caacatcacg ccggagttct tcgtacaggt

7921 cggggccttc gagcagaacc cttccaacct ggaaaccggc aacggcttca agctcagcgg

7981 cagtggtacc aagggggcga tcttgccggt ggaagcggtg tggtcgccca aggtcaatgg

8041 cctgccgggc gagtaccgcc tgggttacta ctacagcacg gccaaggctg acgatgtgta

8101 cgacgacgtc aacggcaacc cgcaggcgct gacaggtgaa gccttcaagt cgcactccag

8161 caagcacgga tggtgggtgg tggcgcagca gcaggtcact gcccatggcg gcgacgtcaa

8221 ccggggcctc agcctgttcg ccaacttcac cgtgcacgac aaggccacca acgtggtcga

8281 caactaccag caggtggggc tggtctacaa aggcgctttc gacgcccggc ccaaggatga

8341 catcggcttc ggcgtggcgc gtattcatgt gaatgacgac gtgaagaagc gcgccgaact

8401 gctcaacgca cagagcggca tcaacgatta cgacaaccct ggtttcgtgc cgctgcagcg

8461 taccgaatac aacgcagagc tctactacgg cttccacgtt accaactggc tgaccgtgag

8521 gcccaacctg cagtacatca agagccctgg cggggtggac gaggtggata acgcgctggt

8581 cgctggcttg aagattcagt cgtcattcta aactagtctt ggactcctgt tgatagatcc

8641 agtaatgacc tcagaactcc atctggattt gttcagaacg ctcggttgcc gccgggcgtt

8701 ttttattggt gagaatccag gggtccccaa taattacgat ttaaatttgt gtctcaaaat

8761 ctctgatgtt acattgcaca agataaaaat atatcatcat gaacaataaa actgtctgct

8821 tacataaaca gtaatacaag gggtgttatg agccatattc agcgtgaaac gagctgtagc

8881 cgtccgcgtc tgaacagcaa catggatgcg gatctgtatg gctataaatg ggcgcgtgat

8941 aacgtgggtc agagcggcgc gaccatttat cgtctgtatg gcaaaccgga tgcgccggaa

9001 ctgtttctga aacatggcaa aggcagcgtg gcgaacgatg tgaccgatga aatggtgcgt

9061 ctgaactggc tgaccgaatt tatgccgctg ccgaccatta aacattttat tcgcaccccg

9121 gatgatgcgt ggctgctgac caccgcgatt ccgggcaaaa ccgcgtttca ggtgctggaa

9181 gaatatccgg atagcggcga aaacattgtg gatgcgctgg ccgtgtttct gcgtcgtctg

9241 catagcattc cggtgtgcaa ctgcccgttt aacagcgatc gtgtgtttcg tctggcccag

9301 gcgcagagcc gtatgaacaa cggcctggtg gatgcgagcg attttgatga tgaacgtaac

9361 ggctggccgg tggaacaggt gtggaaagaa atgcataaac tgctgccgtt tagcccggat

9421 agcgtggtga cccacggcga ttttagcctg gataacctga ttttcgatga aggcaaactg

9481 attggctgca ttgatgtggg ccgtgtgggc attgcggatc gttatcagga tctggccatt

9541 ctgtggaact g

**Sequence S2.** Multiple alignment of OprB amino acid sequences from *P. putida* EM42 using CLUSTALW (fast pairwise alignment; word size 1, window size 5, gap penalty 3). Conserved residues identified by ESPript 3.2 (Gouet et al. 1999) are highlighted by red background, similar residues (global score 0.6) are in red and framed in blue. Protein similarity based on the amino acid level calculated by blastp: OprB-I and OprB-II 71.2 % (cover 100 %), OprB-I and OprB-III 32.2 % (cover 97 %), OprB-II and OprB-III 32.1 % (cover (98 %).


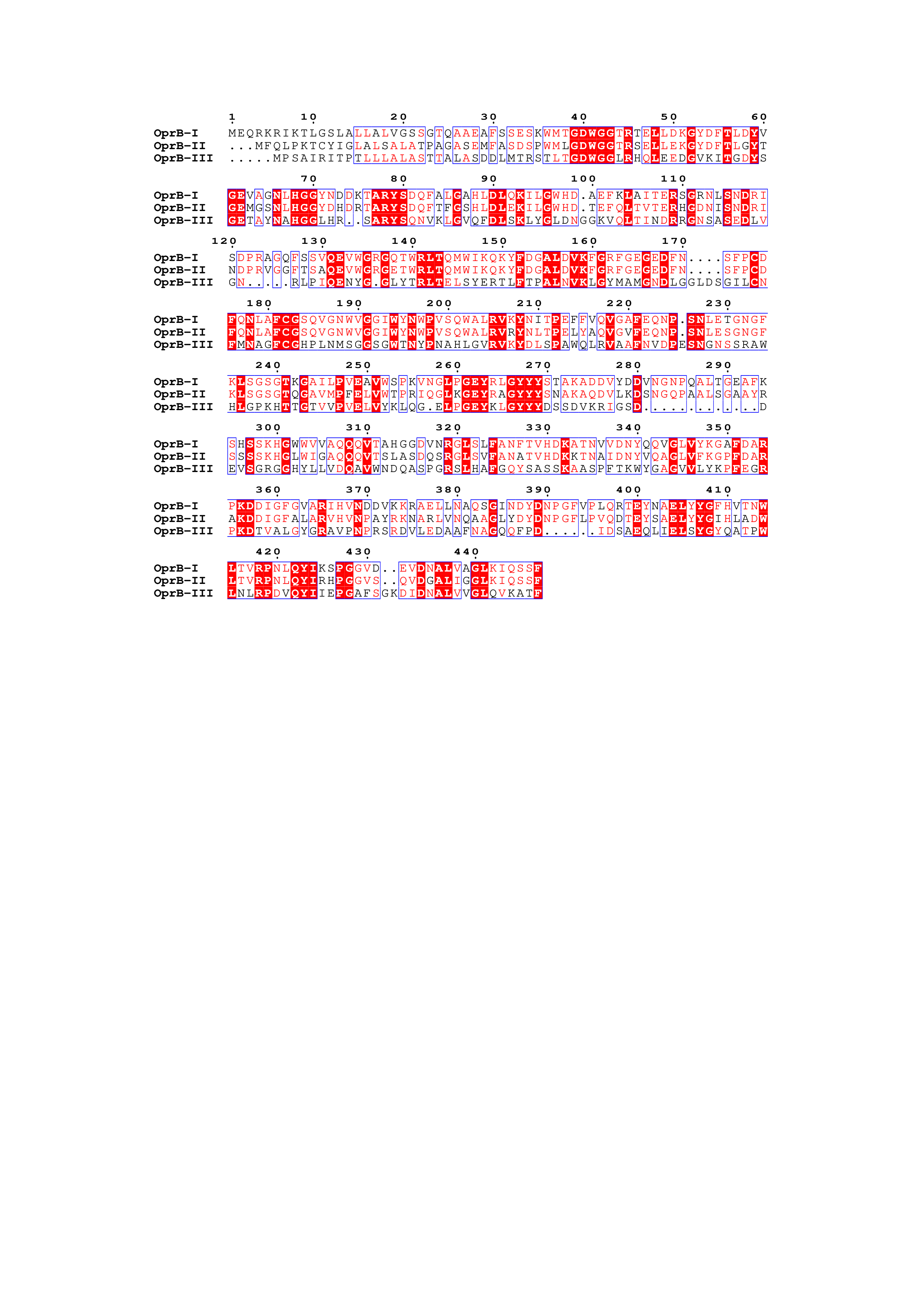


**Supplementary references**

Gouet, P., E. Courcelle, D. I. Stuart, and F. Metoz. 1999. “ESPript: Analysis of Multiple Sequence Alignments in PostScript.” Bioinformatics 15 (4): 305–8. https://doi.org/10.1093/bioinformatics/15.4.305.
